# The transcriptional regulation of the horizontally acquired iron uptake system, yersiniabactin and its contribution to oxidative stress tolerance and pathogenicity of globally emerging *salmonella* strains

**DOI:** 10.1080/19490976.2024.2369339

**Published:** 2024-07-04

**Authors:** Imbar Diamant, Boaz Adani, Meir Sylman, Galia Rahav, Ohad Gal-Mor

**Affiliations:** a The Infectious Diseases Research Laboratory, Sheba Medical Center, Tel-Hashomer, Israel; b Faculty of Medicine, Tel Aviv University, Tel Aviv, Israel; c Department of Clinical Microbiology and Immunology, Tel Aviv University, Tel Aviv, Israel

**Keywords:** *Salmonella* Infantis, *Salmonella* Muenchen, pESI, yersiniabactin, virulence, Ybt, iron acquisition, pathogenicity, gene regulation, emerging pathogens, siderophore

## Abstract

The bacterial species *Salmonella enterica* (*S. enterica*) is a highly diverse pathogen containing more than 2600 distinct serovars, which can infect a wide range of animal and human hosts. Recent global emergence of multidrug resistant strains, from serovars Infantis and Muenchen is associated with acquisition of the epidemic megaplasmid, pESI that augments antimicrobial resistance and pathogenicity. One of the main pESI’s virulence factors is the potent iron uptake system, yersiniabactin encoded by *fyuA, irp2-irp1-ybtUTE, ybtA*, and *ybtPQXS* gene cluster. Here we show that yersiniabactin, has an underappreciated distribution among different *S. enterica* serovars and subspecies, integrated in their chromosome or carried by different conjugative plasmids, including pESI. While the genetic organization and the coding sequence of the yersiniabactin genes are generally conserved, a 201-bp insertion sequence upstream to *ybtA*, was identified in pESI. Despite this insertion, pESI-encoded yersiniabactin is regulated by YbtA and the ancestral Ferric Uptake Regulator (Fur), which binds directly to the *ybtA* and *irp2* promoters. Furthermore, we show that yersiniabactin genes are specifically induced during the mid-late logarithmic growth phase and in response to iron-starvation or hydrogen peroxide. Concurring, yersiniabactin was found to play a previously unknown role in oxidative stress tolerance and to enhance intestinal colonization of *S*. Infantis in mice. These results indicate that yersiniabactin contributes to *Salmonella* fitness and pathogenicity *in vivo* and is likely to play a role in the rapid dissemination of pESI among globally emerging *Salmonella* lineages.

## 1. Introduction

The bacterial species *Salmonella enterica* (*S. enterica*) is a Gram-negative, highly ubiquitous pathogen that can infect a wide range of animal and human hosts.^1^ Humans infection with non-typhoidal *Salmonella* serovars (NTS) causes, in most cases, a localized self-limiting inflammation of the terminal ileum and colon known as gastroenteritis. However, infection with typhoidal *Salmonella* serovars such as *S*. Typhi or *S*. Paratyphi A leads to a systemic life-threatening disease, called typhoid or enteric-fever (reviewed in).^2,3^

Amongst more than the 2,600 serovars known, *S*. *enterica* serovar Infantis (*S*. Infantis) is one of the prevalent serovars worldwide, and emergence of multidrug resistant (MDR) *S*. Infantis strains was reported during the last decade in multiple countries around the globe.^4^ Previously, we showed that the first identified emergence of an MDR *S*. Infantis strain in Israel was facilitated by horizontal acquisition of a unique virulence-resistance megaplasmid, named pESI (standing for plasmid of emerging *S*. Infantis), and demonstrated that pESI acquisition enhances *Salmonella* tolerance to oxidative stress and mercury toxicity, and augments biofilm formation and pathogenicity in the mouse model,^5–7^ Moreover, we showed that pESI is a conjugative plasmid that can be disseminated *in vivo* to different microbiota species and other pathogens.^8^ Subsequently, pESI and genetically related pESI-like plasmids were further identified in many emerging *S*. Infantis lineages worldwide,^9–16^ These pESI-like plasmids share the same backbone but may carry varying antibiotic resistant cassettes, including extended-spectrum β-lactamase (ESBL) genes.^9,17–20^

Importantly, *S*. Infantis is not the only serovar whose global emergence was linked to pESI acquisition. Recently, we reported the rapid emergence of an MDR *S*. *enterica* serovar Muenchen (*S*. Muenchen) strain in Israel, which was also associated with the acquisition of pESI, and further identified similar pESI-positive clinical isolates of *S*. Muenchen in the USA, UK, and South Africa.^21^ Moreover, Dos Santos and colleagues have reported the identification of pESI in additional *Salmonella* serovars including *S*. Schwarzengrund, *S*. Agona, and *S*. Senftenberg.^22^ Collectively, these reports indicate worldwide dissemination of epidemic *Salmonella* strains harboring pESI or pESI-like megaplasmids that are presumed to play an important role in the evolution, dissemination, and pathogenicity of their bacterial host strains.

In addition to variable presence of antibiotic resistance genes conferring the MDR phenotype, pESI encodes several other virulence and defense factors, including the Klf and Ipf chaperon-usher fimbriae,^23^ the mercury resistance *mer* operon and the potent iron acquisition system, yersiniabactin.^5,7^

Although iron is one of the most abundant elements on Earth, at its oxidized ferric (Fe^3+^) form it is hardly available to bacteria under aerobic conditions (10^−17^ M solubility at pH ~ 7). Inside the mammalian and avian hosts, Fe^3+^ concentration is even lower and reaches 10^−18^ M, due to its sequestering by host iron-binding proteins such as transferrin, lactoferrin, hemoglobin, and ferritins. These ferric concentrations are far below the equilibrium concentration required for the optimal growth of microbes (10^−7^ to 10^−5^ M),^24,25^ leading to a host-directed condition known as “nutritional immunity”.^26^ Therefore, to obtain iron, bacteria have developed efficient iron acquisition systems called siderophores, which are used to scavenge ferric from the environment or the host under iron-limited conditions. Siderophores are low molecular weight ferric iron-specific chelators, which are produced and secreted by bacteria, allowing bacterial pathogens to compete with the high-affinity iron-binding host transferrin and lactoferrin.^27^ After the binding of the iron to the siderophores, they are transported back to the bacteria via outer membrane ferri-siderophores receptors (such as FepA and FhuA) that bind cognate ferri-siderophores with high specificity.^28^

As a highly iron-dependent intracellular pathogen, *S. enterica* synthesizes several catechol siderophores including enterobactin and salmochelin.^29,30^ Another type of a siderophores, found in Enterobacteriaceae is the yersiniabactin (Ybt). This iron uptake system enables iron scavenging capabilities upon host infection and is biosynthesized by a nonribosomal peptide synthetase. The yersiniabactin biosynthetic (*ybtT, ybtE, ybtS*), transport (*fyuA, ybtP, ybtQ*), and regulatory (*ybtA*) genes are clustered on a mobile genetic element, known as the High Pathogenicity Island (HPI) that is required for the virulence of high-pathogenic *Yersinia* species in mice.^31^ The HPI is also responsible for the dissemination of the *ybt* genes among virulent strains of *Citrobacter, Enterobacter, Klebsiella, Photorhabdus, Serratia*, and *E. coli*.^32^ Iron scavenging activity by Ybt involves several steps including (i) the binding of a ferric iron, (ii) its active transport by the outer membrane via a TonB-dependent β barrel protein (FyuA), and (iii) delivery of the iron to the cytosol through the ATP cassette proteins (YbtPQ).^31^

While the presence of yersiniabactin in pESI has been previously reported by us and others in *S*. Infantis^5,9^ and *S*. Muenchen,^21,22^ the contribution of this system to *Salmonella* fitness and pathogenicity and the way pESI-encoded yersiniabactin is regulated in *Salmonella* remained unknown. These aspects were therefore studied and are reported below.

## 2. Materials and methods

### 2.1. Bacterial strains and growth conditions

All bacterial strains and plasmids used in this study are listed in Table S1. The wildtype (WT) *S*. Infantis strain 119944^6^ and its isogenic mutant strains were grown in 2 ml LB-Lennox broth supplemented with 20 μg/ml tetracycline at 37°C on a roller drum. Growth in minimal medium was conducted in M9 [72 mM Na_2_HPO_4_, 22 mM KH_2_PO_4_, 8.55 mM NaCl, 18.7 mM NH_4_Cl,100 mM Trizma base, 2 mM MgSO_4_, 0.1 mM CaCl_2_, 11.1 mM glucose] (pH = 7.4). For iron depletion growth conditions, cultures grown in LB were washed with M9 and subcultured by dilution 1:30 or 1:100 into fresh M9 or LB media in the presence of the iron chelator 2, 2′-dipyridyl (DIP) at varying concentrations (50, 100, 200, or 400 μM).

### 2.2. *Salmonella* tolerance to oxidative stress

The wildtype *S*. Infantis strain 119944 and its isogenic *irp2* mutant were grown overnight in 2 ml LB broth supplemented with 20 μg/ml tetracycline at 37°C. One ml of overnight cultures were centrifuged at 10,000 g for 2 min, washed with 1 ml of M9 minimal medium, diluted 1:30 into 2 ml fresh M9 media and grown aerobically at 37°C for 4–5 h until reaching an OD_600_ ~1. Hydrogen peroxide (Merck) at final concentration of 4, 20, or 40 mM was added to the cultures and incubated at 37°C for 10, 20, or 30 min. Serial dilutions that were made in saline (0.9% NaCl) and plated on selective (tetracycline) LB agar plates were used for CFUs counting.

### 2.3. RNA purification and quantitative real-time reverse transcription – polymerase chain reaction (qRT-PCR)

*Salmonella* cultures were grown overnight in 2 ml LB-Lennox broth supplemented with 20 μg/ml of tetracycline at 37°C under aerobic conditions, diluted 1:100 into fresh LB broth and incubated for about 3 h until cultures reached the late logarithmic phase (OD_600_ ~1.0). For cultures grown in M9 minimal medium, overnight cultures were washed, subcultured in fresh M9 medium and grown for 8 h under the same conditions. For RNA extractions, *Salmonella* cultures were mixed in a 1:2 ratio with RNA Protect Bacteria Reagent (QIAGEN) and RNA was extracted using the RNeasy mini kit (QIAGEN), according to the manufacturer’s protocol. RNA concentration was measured using Nanodrop 2000c (Thermo Fisher Scientific) and treated with RNase-free DNase (QIAGEN). cDNA was synthesized using qScript cDNA Synthesis Kit (Quanta-bio) in a T100 thermal cycler (Bio-Rad). Each reaction was carried out in a total volume of 20 μl in a 96-well optical reaction plate (Applied Biosystems) containing 10 μl FastStart Universal SYBR green Master mix, 2 μl cDNA, and target gene-specific primers at a final concentration of 0.3 μM each. Melting-curve analysis verified that each qRT-PCR reaction has generated a single amplimer. Relative quantification of transcripts was determined using the comparative threshold cycle (CT) method. Transcript levels were normalized to the housekeeping genes *rpoD* or 16S rRNA. The Δ*CT* values were calculated by determining the difference in threshold values for the target gene and the normalized gene in the WT versus the mutant background, or for the control versus the experimental growth condition. The ΔΔ*CT* value was calculated by subtraction of the normalized Δ*CT* value in the wildtype strain (or control condition) from the normalized Δ*CT* value of the compared mutant (or the experimental condition).

### 2.4. Cloning and mutants construction

All primers used in this study are listed in Table S2. Oligonucleotides were purchased from IDT, and PCR was carried out using the Phusion Hot Start Flex DNA Polymerase (New England BioLabs). In-frame deletion of all *S*. Infantis mutants was constructed using the λ-red-recombination system. One-step in-frame deletion of the genes *ybtA*, and *iroB* using the primer pairs ‘ybtA-KO_Fw’ and ‘ybtA-KO_RV’; and ‘iroB-KO_Fw’ and ‘iroB-KO-Rv’, respectively, was constructed according to the protocol previously described.^33^ Gene knockout by the λ red recombinase system using a 3-steps PCR method^34^ was used to construct an in-frame deletion of *S*. Infantis 119944 *entC* mutant using the primer pairs ‘P1- entC KO’ and ‘P2- entC KO’; and ‘P3- entC KO’ and ‘P4- entC KO.’ To produce an amplimer containing the antibiotic resistance cassette, PCR was done in a 50 μl reaction volume with the primers listed in Table S2. The PCR products were gel purified using the QIAEX Gel Extraction kit (QIAGEN). pKD46 was transformed by electroporation into *S*. Infantis 119944 and ampicillin-resistant colonies were selected on selective LB plates at 30°C. Linear DNA containing the inactivated target genes interrupted by a kanamycin-resistance cassette were introduced into *S*. Infantis 119944 strain harboring pKD46 by electroporation following induction with 20 mM L-arabinose. Excision of the antibiotic marker was done by an FLP-mediated recombination, using the curable temperature-sensitive plasmid pCP20. All obtained mutants were verified by sanger sequencing using flanking primers of the target genes.

To complement the *irp2* deletion (*Δirp2)* in *S*. Infantis, a DNA fragment containing the entire *irp2* gene and its upstream regulatory region was PCR-amplified from *S*. Infantis 119944 genome using the GoTaq Long PCR Master Mix (Promega) and the primers ‘irp2 fw Hind3’ and ‘irp2 rev BamH1’. The resulted 6405-bp amplimer was digested with BamHI and HindIII and cloned into the low copy number vector pWSK29 using a T4 ligase (Promega).

To complement the *ybtA* deletion (*ΔybtA*) in *S*. Infantis, a DNA fragment containing the entire *ybtA* gene and its upstream regulatory region was PCR-amplified using *S*. Infantis 119944 gDNA as a DNA template and the primers ‘ybtA_fwd_Comp’ and ‘ybtA_rev_Comp’. The resulted 1398-bp PCR product was digested with BamHI and NotI and cloned into pWSK29.

To complement the *S*. Infantis *entC* null deletion (*ΔentC)*, a DNA fragment containing the complete *entC* gene and its upstream regulatory region was PCR-amplified using *S*. Infantis 119944 gDNA as a DNA template and the primers ‘entC_COMP_FW’ and ‘entC_COMP_REV.’ The obtained 1508-bp amplimer was digested with BamHI and SacI and cloned into pUC18. Subcloning using the same restriction enzymes was subsequently used to clone the insert in pWSK29.

### 2.5. A bioluminescence reporter system for the ybtA promoter

For the construction of a *ybtA* bioluminescence reporter system, the regulatory region of *ybtA* containing 367-bp upstream from it’s start codon was amplified with the primers ‘Fw ybtA prom pCS26’ and ‘Rev ybtA prom pCS26,’ using *S*. Infantis 119944 gDNA as a DNA template. The obtained PCR fragment was digested with XhoI and BamHI, and cloned into pCS26 to generate the reporter plasmid pCS26::P*ybtA*. This construct expresses the *luxCDABE* operon of the bacterium *Photorhabdus luminescens*^35^ under the regulatory region of the pESI-encoded *ybtA* gene. Promoter activity in *S*. Infantis cultures was measured with the Infinite 200 Pro M-PLEX microplate reader (TECAN) at 37°C under regular shaking in a white 96-well flat bottom plate (Greiner), using the luminescence mode. Absorbance was read at 600 nm.

### 2.6. Rapid amplification of cDNA ends (5’−RACE)

To identify the transcriptional start site of *ybtA*, the SMARTre RACE 5’/3’ kit (Takara Bio USA, Inc.) was used. Total RNA was extracted from a subculture of *S*. Infantis grown in LB supplemented with 200 µM DIP at 37°C under aerobic conditions to an OD_600_ of ~1.5, using RNeasy Mini kit (QIAGEN) after RNAprotect bacteria Reagent (QIAGEN) treatment. 5’-RACE-Ready cDNA synthesis was conducted using 1 µg *S*. Infantis RNA and 1.0 μl of 10× Random Primer Mix according to the manufacturer instructions. 5’- RACE PCR reaction was conducted in 50 μl reaction volume containing 2.5 µl 5’-RACE-Ready cDNA and a *ybtA* gene-specific primer using the following PCR program: 30 cycles of 94°C 30 sec, 68°C 30 sec, 72°C 3 min. The obtained 1.1 kb PCR product was separated and purified from an agarose gel using the NucleoSpin Gel and PCR Clean-up (MACHEREY-NAGEL) kit. Gel-purified RACE product was then cloned using In-Fusion Snap Assembly and electrophorated into *E. coli* HST08 competent cells that were platted onto ampicillin-selective LB agar plates. Ten independent colonies were picked up, plasmid purified, and Sanger sequenced using the primer ‘ybtA middle Rv’.

### 2.7. Expression and purification of his-tagged Fur

The entire Fur coding region was amplified by PCR from the genome of *S*. Infantis 119944 using the primer pair ‘fur for pet28a Nde1 Fw’ and ‘fur BamH1 Rv- new’ (Table S2) and cloned into the expression plasmid pET28a, between BamHI and NdeI sites, generating an N-terminal 6×His tag fusion. The recombinant plasmid pET28a::*fur* was introduced into *E. coli* BL21 (DE3) cells. His-tagged Fur was purified from 3 ml culture that were grown in LB medium containing 20 μg/ml kanamycin at 37°C for 2 h with vigorous shaking, until OD_600_ reached 0.6–0.8. Expression was induced by adding IPTG to a final concentration of 1 mM, and incubation for an additional 4 h. Cells were harvested by centrifugation for 1 min at 14,000 g, resuspended in 500 μl of commercial MagListo Binding/Washing buffer, and then sonicated on ice using a XL2020 Sonicator (Heat Systems) equipped with a micro-tip. The lysate was centrifuged at 14,000 g for 10 min at 4°C. *S*. Infantis 6× His-tagged Fur protein was purified using the MagListo His-tagged Protein Purification Kit (Bioneer) according to the kit protocol. Briefly, following sonication, 500 μl of the cleared lysate was loaded onto pre-equilibrated Ni-NTA magnetic silica resin that was placed on the magnetic bar for 1 min and washed with MagListo Binding/Washing buffer three times. For protein elution, 500 μl of Elution Buffer was added, and the eluted fractions were collected and analyzed on 15% polyacrylamide gel electrophoresis (SDS – PAGE).

### 2.8. Electrophoretic mobility shift assay (EMSA)

The primers ‘ybtA pro_119944_Fw’ and ‘ybtA pro_SIN+yersi_Rv’ were designed to PCR amplify a 387-bp promoter proximate region extending upstream from the start codon of the *ybtA* gene in pESI. DNA binding was tested in a 10 μl reaction volume containing binding buffer [100 μM MnCl_2_, 1 mM MgCl_2_, 0.5 mM dithiothreitol (DTT), 50 mM KCl, 10 mM Tris-HCl (pH 7.5), 0.05 mg of bovine serum albumin/ml, and 4% glycerol], the DNA probe (150 ng) and increasing amounts of purified 6×His-tagged Fur. After incubation at room temperature for 30 min, the DNA-protein complex was loaded onto a native 4% polyacrylamide gel and electrophoresed in 0.5×Tris-borate buffer containing 100 μM MnCl_2_ for 30 min at constant voltage of 220 V.

### 2.9. Macrophages infection in vitro

The human macrophage cell line U937 was purchased from American Type Culture Collection (ATCC) and mouse bone-marrow-derived macrophages (BMDMs) were isolated from the femur leg bone of 7-week-old SWISS female mice according to.^36^ Briefly, macrophages were seeded at 0.5 × 10^6^ cells/ml, in a 24-well tissue culture dish, 24 h prior to bacterial infection, and infected at multiplicity of infection (MOI) of 1:10. Macrophages were infected with cultures of *S*. Infantis 119944 wildtype and the Δ*irp2* mutant strain, while *S*. Typhimurium SL1344 wildtype and its Δ*ssaR* isogenic mutant were included as positive and negative controls, respectively. Infection experiments were carried out using the gentamicin protection assay as described in.^37^ Briefly, overnight *Salmonella* cultures were centrifuged and resuspended in DMEM or BMDM [50% DMEM high glucose, 20% FBS, 30% L-929 conditioned medium, 2 mM L-glutamine, 1 mM sodium pyruvate, 50 nM β-mercaptoethanol] for U937 and BMDMs infection, respectively. The cells were infected with 1 ml culture from each strain and incubated for 1 h at 37°C under 5% CO_2_ atmosphere. Each well was washed three times with PBS to remove extracellular bacteria, and medium containing 100 μg/ml gentamicin was added for 1 h incubation. Wells were then washed three times with PBS, and medium was replaced with fresh DMEM or BMDM containing 10 μg/ml gentamicin. To determine intracellular growth of *Salmonella* at 2 and 24 h post infection (p.i.), cells were washed three times with PBS and lysed with 250 μl of lysis buffer (0.1% SDS, 1% Triton X-100 in PBS). The number of CFUs in each well was quantified by plating serial dilutions of cell lysates on selective LB-agar plats. *Salmonella* replication was calculated as the number of intracellular bacteria recovered at 24 h p.i., divided by intracellular bacteria recovered at 2 h p.i.

### 2.10. Competitive index (C.I.) infection in mice

All *in vivo* experiments were conducted according to the ethical requirements of the Animal Care Committee of the Sheba Medical Center (Approval # 1182/18) and in line with the national guidelines of the Animal Testing Council. Female C57BL/6 mice (Envigo, Israel) were infected at an age of 8 weeks as previously described.^23^ Briefly, Streptomycin (20 mg per mouse) was given by an oral gavage in saline 24 h prior to infection. Mice were orally infected with 6 × 10^6^ CFU of 1:1 mix of *S*. Infantis 119944 WT (containing pWSK129 conferring kanamycin resistance), and its isogenic *irp2* mutant strain (harboring pWSK29 conferring ampicillin resistance). Three days p.i. the cecum and colon were harvested, aseptically homogenized in 750 μl of saline, and plated on selective XLD agar plates supplemented with ampicillin or kanamycin. CFUs were counted following 24 h incubation at 37°C. The CI was calculated as [*irp2*/WT] _output_/[*irp2*/WT] _input_.

## 3. Results

### 3.1. Distribution and genetic organization of the yersiniabactin genes among Salmonella strains

**Figure 1.**
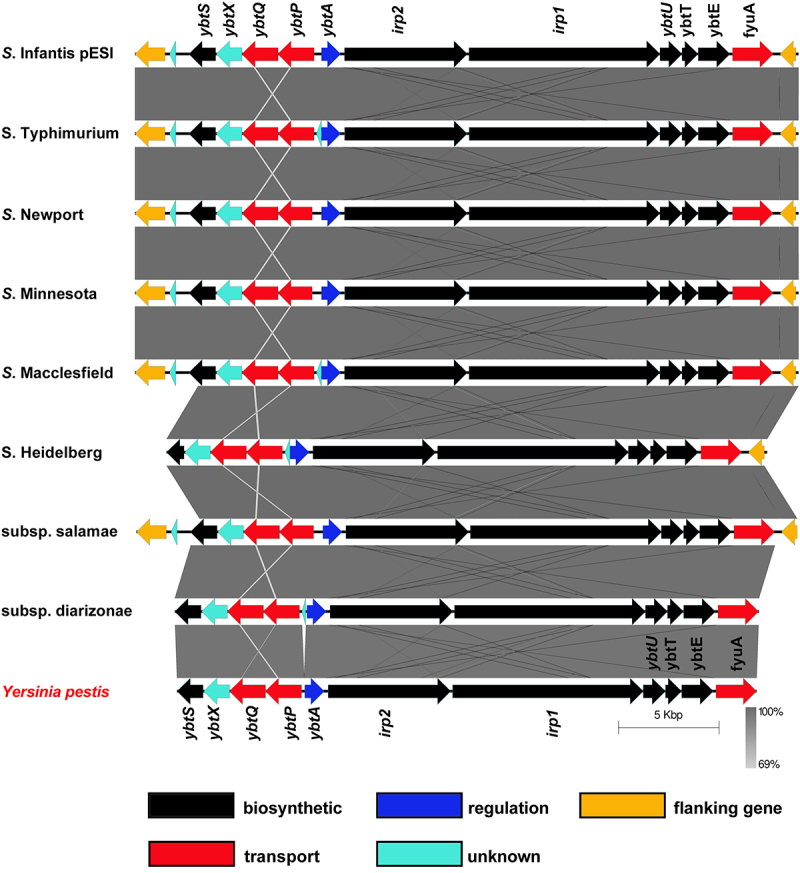
The distribution and genetic organization of the yersiniabactin cluster among *S.* *enterica* subspecies and serovars. DNA sequence and gene organization of the yersiniabactin locus in S. Infantis pESI (accession number CP047882) was compared to its homologous regions in plasmid pSC-31-2 of S. Typhimurium var. 5 (accession number: CP028316), plasmid pCFSAN024415 of S. Newport (accession number: CP074336), plasmid pSA18578_2 of S. Minnesota (accession number: CP080515), unnamed plasmid of S. Macclesfield (accession number: CP022118), plasmid p3 of S. Heidelberg (accession number: CP031362), plasmid pCFSAN001016 of *S.* *enterica* subsp. salamae (accession number: CP074595), the chromosome of *S.* *enterica* subsp. diarizonae (accession number: CP023345), and the HPI region in *Yersinia pestis* strain SCPM-O-B-5942 (accession number: CP045258). Genetic comparison and graphical illustration of the yersiniabactin genes was prepared using the EasyFig tool (https://mjsull.Github.io/Easyfig/). The degree of sequence similarity was computed by BLAST and is shown by shades of gray. Yersiniabactin biosynthetic genes are colored in black, transport genes in red, regulatory gene in blue and genes with unknown function are illustrated in turquoise. The genes flanking the integration site of the *ybt* cluster are shown in yellow. Supplementary information indicating the degree of identity between the *Salmonella* Ybt proteins and their homologs in *Y.* *pestis* is provided in Table S3.

Previously, we reported that the epidemic plasmid, pESI that is associated with emerging strains of *S*. Infantis and *S*. Muenchen carries the entire yersiniabactin cluster.^5,6,21^ Nucleotide Blast (BLASTN) analysis using the yersiniabactin cluster from *Y. pestis* strain SCPM-O-B-5942 (accession number CP045258) against *Salmonella enterica* (taxid:28901) database revealed that a conserved and complete *ybt* cluster, similar to the one carried by pESI is also integrated in other conjugative megaplasmids (size >100-Kbp) found in different isolates of *S. enterica* subsp. enterica serovars Typhimurium var. 5 (e.g. plasmid pSC-31-2 of strain CFSAN067217; accession number: CP028316), Newport (e.g. plasmid pCFSAN024415 of strain CFSAN024415; accession number: CP074336), Minnesota (e.g. plasmid pSA18578_2 of strain SA18578; accession number: CP080515), Macclesfield (e.g. unnamed plasmid of strain S-1643; accession number: CP022118), Reading (plasmid pN18S2042 of strain CVM N18S2042; accession number: CP082537.1), and Heidelberg (e.g. plasmid p3 of strain 5; accession number: CP031362). In these plasmids the *ybt* cluster is inserted between a PhoPQ-activated, PqaA family protein (protein ID WP_020833669.1) and an N-acetylmuramoyl-L-alanine amidase (protein ID WP_020833656.1) encoded genes. In addition, a *ybt* cluster was found integrated in a 173 kb plasmid (pCFSAN001016) of *S. enterica* subsp. salamae (Subsp. II) strain CFSAN001016 (accession number: CP074595) and in the chromosome of multiple isolates of *S. enterica* subsp. diarizonae (Subsp. IIIb) (e.g. strain HZS154; accession number: CP023345) as shown in Figure 1. tBLASTn analysis indicated high conservation with an amino acid sequence identity that ranged between 97% and 99% between the *Y. pestis* Ybt proteins and their homologs in the different *Salmonella* strains (Table S3). These data indicate that the yersiniabactin cluster has a variable and underappreciated distribution in the genome of at least three subspecies of *S. enterica* (enterica, salamae, and diarizonae) and that it can be harbored either on their chromosome or on large conjugative plasmids, which facilitates the dissemination of the yersiniabactin genes horizontally.^8^

### 3.2. Ybt is induced, but not required for the growth of S. Infantis under iron-depleted conditions

**Figure 2.**
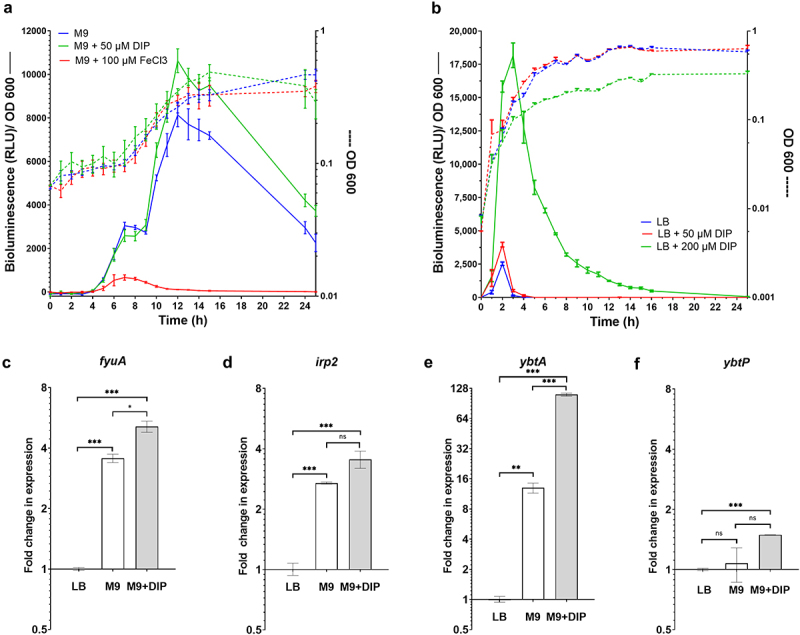
*ybt* genes are induced in S. Infantis pESI during the mid-late logarithmic phase and under iron-limiting conditions. (a) S. Infantis 119944 expressing the *luxCDABE* operon under the *ybtA* pESI promoter (*ybtA::lux*) was grown for overnight in selective LB medium at 37°C, washed and subcultured (1:30) into minimal M9 medium (blue line), M9 medium supplemented with 50 µM of the iron chelator 2,2-dipyridyl (DIP) (green line), and M9 supplemented with 100 µM FeCl_3_ (red line) in a 96-well microplate. The optical density of the culture at 600 nm (right Y-axis and broken lines) and the bioluminescence signal normalized to OD_600_, shown as relative light units (RLU; left Y-axis and continuous lines) were measured for 24 h using a Tecan plate reader set to 37°C with shaking. The chart shows the mean and standard deviation (SD) of six replicates in one (out of three) representative experiment. (b) The same experiment was conducted following subculture of S. Infantis 119944 strain carrying the *ybtA::lux* reporter construct in LB medium (blue line), LB supplemented with 50 (red line), or 200 (green line) µM DIP. The chart shows the mean and SD of three replicates in one (out of three) representative experiment. (C-F) The transcription of *fyuA* (c), *irp2* (d), *ybtA* (e) and *ybtP* (f) was determined using qRT-PCR for S. Infantis 119944 cultures grown at 37°C to the late logarithmic phase (OD_600_ 0.8–1) in M9 minimal medium or M9 supplemented with 400 µM DIP relative to their expression in LB broth. The fold change in expression is shown as the mean and standard error of the mean (SEM) of at least three biological repeats. One-way ANOVA was used to determine statistical significance between gene expression in LB and the expression in M9 or M9+DIP. Unpaired t-test was used to compare statistical significance between gene expression in M9 vs. M9+DIP. ns, not significant; *, p < 0.05; **, p < 0.01; ***, p < 0.005.

The expression of most of the iron acquisition systems in *Salmonella* including enterobactin and salmochelin is known to be induced under iron-limiting conditions.^38^ To test if growth under iron depleted conditions affects gene expression of the *ybt* genes in *S*. Infantis, we have constructed a bioluminescence reporter system by cloning the promoter of the yersiniabactin regulator encoded gene, *ybtA* upstream to the *luxCDABE* operon in the plasmid pCS26. This reporter plasmid was introduced into *S*. Infantis and the expression of *ybtA::lux* was studied under different growth conditions. As shown in Figure 2, the *ybtA::lux* expression was highly induced during the mid-late logarithmic growth phase of *S*. Infantis in low-iron M9 minimal medium or in M9 depleted from iron in the presence of the high-affinity iron chelator, 2,2-dipyridyl (DIP). In contrast, adding ferric iron (FeCl_3_) to M9 medium sharply suppressed *ybtA::lux* expression, without affecting its growth (Figure 2(a)). Similarly, *ybtA:lux* expression was suppressed in LB medium, but was greatly induced during the mid-late logarithmic growth phase of the culture, in the presence of 200 µM DIP added to the LB (Figure 2(b)). We concluded from these experiments that *ybtA* promoter is expressed from pESI and induced in *S*. Infantis in the absence of iron.

Previously it was shown that the *ybt* locus is organized into four independent operons composing the genes *fyuA* (termed *psn* in *Y. pestis*), *irp2-irp1-ybtUTE*, *ybtA*, and *ybtPQXS*.^32^ Therefore, to further study the regulation of the *ybt* cluster in *S*. Infantis directly, qRT-PCR was conducted to determine the fold change in the transcription of *fyuA, irp2, ybtA*, and *ybtP* (as the first genes in each operon), during growth in rich LB medium, M9 minimal defined medium, and M9 supplemented with DIP. These results agreed with the bioluminescence reporter system results and showed that in the absence of iron, *fyuA* (Figure 2(c)), *irp2* (Figure 2(d)) and most prominently, *ybtA* (Figure 2(e)) were significantly induced in M9 medium or in M9 supplemented with DIP in comparison to their expression in LB medium. The expression of *ybtP* was rather constant under these conditions and demonstrated only minor, but statistically significant induction in M9 supplemented with DIP (Figure 2(f)). Together, we concluded from these experiments that *ybt* genes in *S*. Infantis pESI are induced *in vitro* during the mid-late exponential growth phase, under iron-limiting growth conditions.

**Figure 3.**
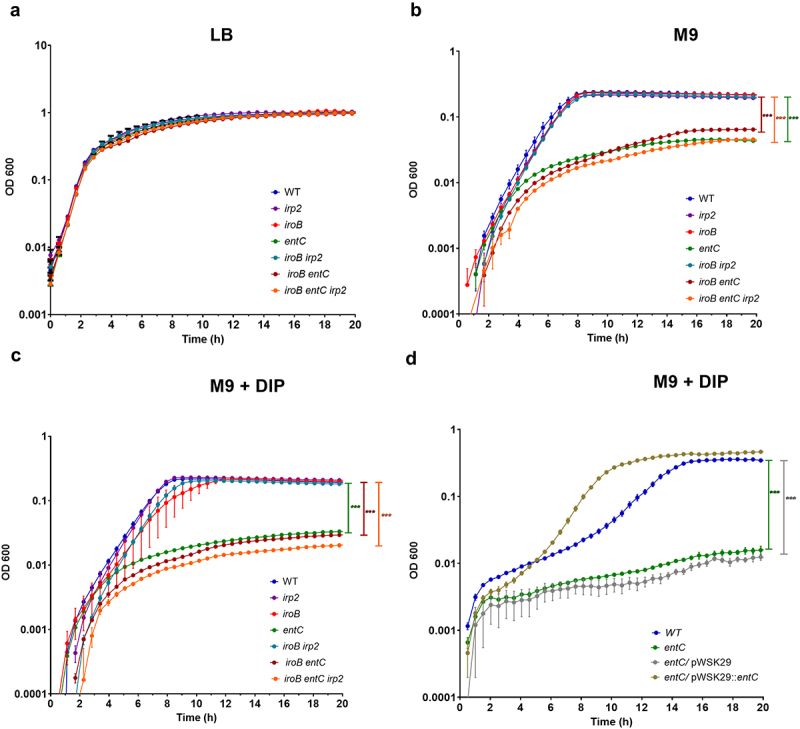
Ybt is not required for S. Infantis growth under iron limiting conditions. S. Infantis 119944 and its isogenic single null mutant strains *irp2* (yersiniabactin), *iroB* (salmochelin), and *entC* (enterobactin); the double mutant strains *iroB irp2* and *iroB entC*; and the triple mutant strain *iroB entC irp2* were grown in LB to their stationary phase at 37°C under aerobic conditions. Subsequently, cultures were washed and subcultured into LB (a), M9 minimal medium (b), or M9 medium supplemented with 100 µM DIP (c). The growth of the strains at 37°C with shaking was determined along 20 h by optical density reading at 600 nm. (d) The growth curves of S. Infantis 119944, its isogenic *entC* null mutant strain and *entC* strains, expressing *entC* from a plasmid (pWSK29::*entC*) or harboring the empty vector (pWSK29) that were grown in M9 medium supplemented with 100 µM DIP as in (c) are plotted. The mean and SEM of 3–5 biological repeats is shown. One-way ANOVA was used to determine statistical significance. ***, p < 0.005.

To determine whether Ybt contributes to *S*. Infantis growth under iron-depletion conditions, we constructed *S*. Infantis mutant strains with null deletions in yersiniabactin (*irp2*), enterobactin (*entC*) and salmochelin (*iroB*). In addition, to account for possible redundancy in these systems, we have constructed the double mutant strains *iroB irp2* and *iroB entC* and a triple mutant strain containing null deletion of all three iron acquisition systems *iroB entC irp2*. The growth of these mutants was compared to the growth of the wildtype *S*. Infantis 119944 parental strain in rich LB medium, in M9 minimal medium and in M9 supplemented with 100 µM DIP. While in rich LB broth, these strains grew similarly (Figure 3(a)), in M9 (Figure 3(b)) and in M9 supplemented with DIP (Figure 3(c)) the growth of the strains containing a null deletion in enterobactin (*entC*) was significantly impaired. Complementing the expression of *entC* from a low copy-number plasmid (pWSK29::*entC*) but not the empty vector itself (pWSK29), restored the growth of the *S*. Infantis Δ*entC* mutant to similar levels as the WT (Figure 3(d)). Moreover, the impaired growth of the *entC* mutant was similar to the growth rate of the double mutant strain *iroB entC* and the triple mutant strain *iroB entC irp2*. These results indicated that while enterobactin contributes significantly to the ability of *S*. Infantis to grow under iron-limiting conditions, salmochelin and yersiniabactin contributed only marginally, if any, to the growth of *S*. Infantis in the absence of iron. Therefore, we thought that the contribution of Ybt to *Salmonella* fitness may lay in a different area.

### 3.3. Ybt is induced in the presence of hydrogen peroxide and contributes to S. infantis oxidative stress tolerance

**Figure 4.**
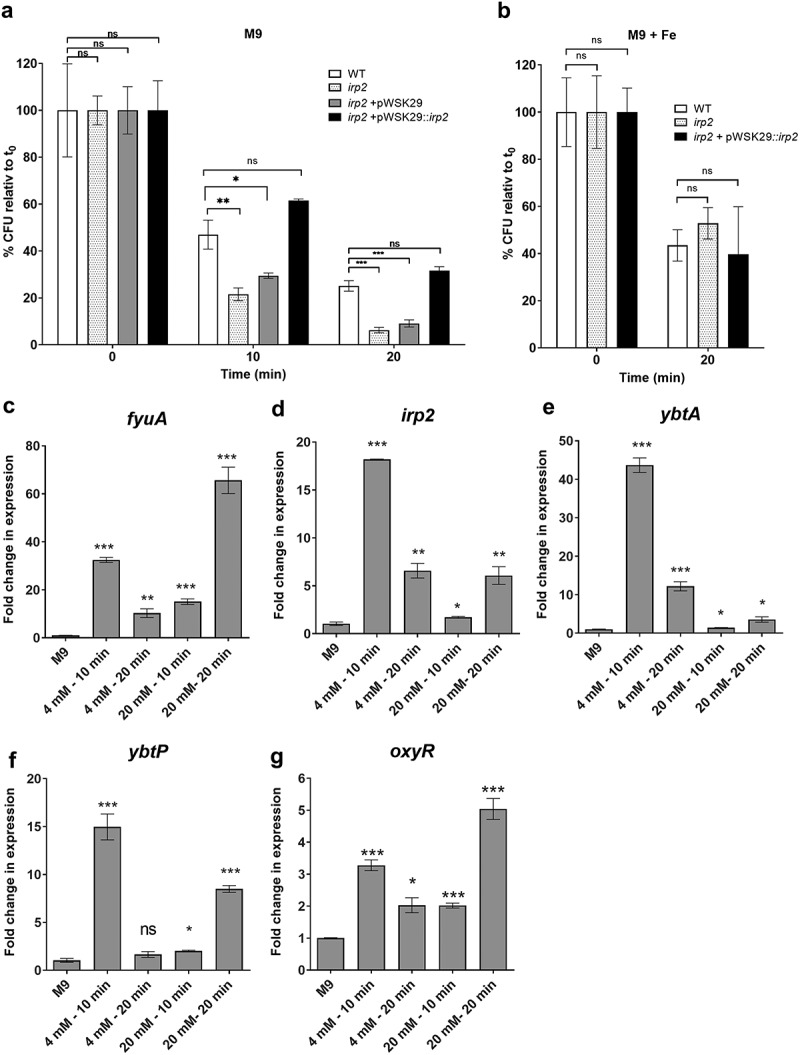
Yersiniabactin is induced in the presence of ROS and contributes to oxidative stress tolerance. (a) *S*. Infantis 119944, its isogenic *irp2* null mutant, and a complemented *irp2* mutant harboring pWSK29::*irp2* or the empty vector (pWSK29) were grown in LB medium, subcultured into M9 medium and grown at 37°C with shaking until reaching OD_600_ of ~ 1. Hydrogen peroxide at a final concentration of 40 mM was added to the cultures, which were further incubated at 37°C. At 10 and 20 min post H_2_O_2_ challenging, serial dilutions were plated on LB agar plates to determine the number of viable CFUs relative to time 0. (b) *S*. Infantis 119944, its isogenic *irp2* null mutant, and a complemented *irp2* mutant harboring pWSK29::irp2 were subcultured in M9 supplemented with 100 µM FeCl_3_ and challenged with hydrogen peroxide as in (a). The number of viable CFUs relative to time 0, following 20 min of hydrogen peroxide (40 mM) challenge is shown. The graphs (a and b) show the mean of three biological repeats with SEM indicated by the error bars. One-way ANOVA was used to test statistical significance. (c-g) The transcription of *fyuA* (c), *irp2* (d), *ybtA* (e), *ybtP* (f) and *oxyR* (g) that was used as a positive control was determined using qRT-PCR. *S*. Infantis 119944 cultures were grown at 37°C to the late logarithmic phase (OD_600_ 0.8–1) in M9 minimal medium or in cultures that were grown in M9 and incubated with 4 or 20 mM hydrogen peroxide for 10 or 20 min. The fold change in expression following hydrogen peroxide exposure relative to expression in M9 is shown as the mean and SEM of three biological repeats. One-way ANOVA was used to determine statistical significance. ns, not significant; *, p < 0.05; **, p < 0.01; ***, p < 0.005.

Catecholate siderophores (enterobactin and salmochelin) were previously shown to display antioxidant qualities and to be involved in protection against reactive oxygen species (ROS) *in vitro*^39^ and intracellularly after macrophage invasion by *S*. Typhimurium.^40^ Moreover, it was demonstrated that enterobactin can chelate intracellular labile iron that is required for neutrophil oxidative responses.^41^ To determine whether the Ybt system contributes to *S*. Infantis oxidative stress tolerance, the survival of the *irp2* mutant strain was compared to its parental wildtype background in the presence of bactericidal concentrations of hydrogen peroxide (H_2_O_2_). Exposure of *S*. Infantis grown in M9 minimal medium to 40 mM H_2_O_2_ followed by CFU counting indicated that the *irp2* mutant strain was significantly more susceptible to oxidative stress than the wildtype strain. Complementing *irp2* expression from a low copy number plasmid (pWSK29::*irp2*) but not the presence of an empty vector (pWSK29) alone in the *irp2* mutant strain restored the tolerance level displayed by the wildtype strain (Figure 4(a)). Similar results were also obtained when cultures were grown in M9 in the presence of 50 µM DIP (Figure S1), suggesting that ferrous ions are not required for the protection activity of Ybt and therefore is likely to be independent of the Fenton reaction. In contrast, conducting this experiment, while cultures were grown in M9 supplemented with 100 mM FeCl_3_ demonstrated similar levels of hydrogen peroxide susceptibility by all backgrounds (Figure 4(b)), most likely due to the suppression of *ybt* genes in the presence of iron as shown in Figure 2. We concluded from these experiments that Ybt contributes to hydrogen peroxide tolerance in *S*. Infantis, under iron-limiting growth conditions, but not when iron is present in excess.

To determine if oxidative stress conditions affect gene expression of the Ybt system, qRT-PCR was used to define the fold change in the expression of *fyuA, irp2*, *ybtA*, and *ybtP. oxyR* that is known to be induced in response to oxidative stress,^42^ was also included in this analysis as a positive control. Fold change in transcription was measured in the presence of H_2_O_2_ relative to gene expression in M9 medium. qRT-PCR results showed that all *ybt* genes were significantly upregulated in the presence of H_2_O_2_, particularly after a 10-min exposure to 4 mM H_2_O_2_, which resulted in 16 to 45-fold induction of *ybt* genes transcription (Figure 4 (c-g)). Collectively, these results indicated that under oxidative stress conditions, pESI encoded *ybt* genes are significantly induced and contribute to the oxidative stress tolerance of *S*. Infantis.

### 3.4. ybt genes expression is coordinated by YbtA and Fur in S. Infantis

**Figure 5.**
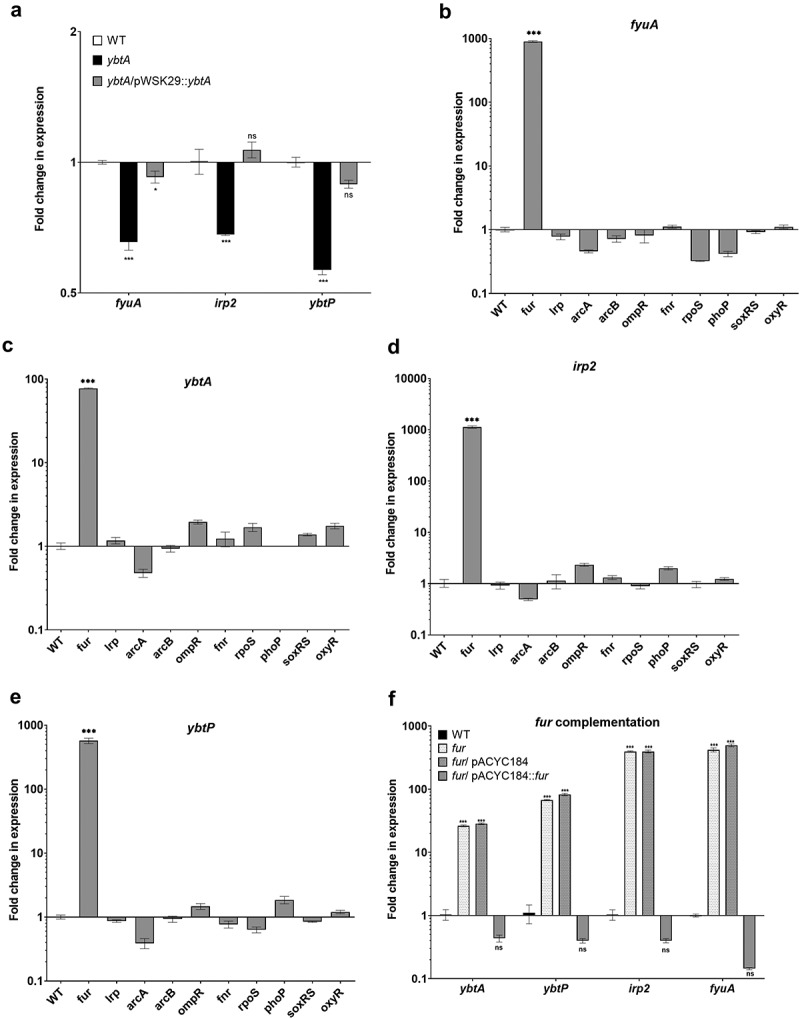
Yersiniabactin gene expression is regulated by YbtA and Fur in *S*. Infantis pESI. (a) *S*. Infantis 119944, its isogenic *ybtA* null mutant strain, and the *ybtA* mutant expressing y*btA* from a plasmid (pWSK::*ybtA*) were subcultured in M9 minimal medium and grown under aerobic conditions at 37°C. RNA that was extracted from late logarithmic phase grown cultures was subjected to qRT-PCR analysis. The relative expression of *fyuA*, *irp2* and *ybtP* in the *ybtA* mutant and the complemented strain relative to their expression in the wildtype (WT) background is shown for three biological repeats. One-way ANOVA was used to determine statistical significance. (b-e) Total RNA was extracted from S. Infantis 119944 wildtype strain and ten isogenic mutants harboring null deletion in key regulatory genes of interest (*fur*, *lrp*, *arcA*, *arcB*, *ompR*, *fnr*, *rpoS*, *phoP*, *soxRS* and *oxyR*) that were grown in LB at 37°C to the late logarithmic phase. qRT-PCR was applied to define the fold change in *fyuA* (b), *ybtA* (c) *irp2* (d), and *ybtP* (e) transcription between the wildtype background and each one of the mutant strains. (f) The expression of *ybtA*, *ybtP*, *irp2*, and *fyuA* was determined in a S. Infantis 119944 (WT), its isogenic *fur* mutant strain (*fur*) and in a *fur* mutant strain complemented with pACYC184::*fu*r or the vector only (pACYC184) relative to their expression in the wildtype background using qRT-PCR. The charts show the mean and SEM of at least three biological repeats. One-way ANOVA was used to test statistical significance. ns, not significant; *, p < 0.05; ***, p < 0.005.

YbtA, is an AraC-like transcriptional regulator, which was previously shown in *Y. pestis* to coordinate Ybt production together with the ferric uptake regulator Fur.^31^ To examine if YbtA plays a similar regulatory role in *S*. Infantis pESI, a null mutation in *ybtA* was constructed in *S*. Infantis 119944. The regulatory role of YbtA in gene expression was tested by qRT-PCR that was applied to define the fold change in *fyuA, irp2* and *ybtP* expression in the *ybtA* null mutant relative to the wildtype background, following growth in M9 minimal medium. These experiments showed that the transcription of the *ybt* genes was subtly decreased by nearly twofold in the absence of YbtA, and that complementing *ybtA* expression from a low copy-number plasmid (pWSK29::*ybtA*) in the Δ*ybtA* mutant strain, resorted the transcription of *fyuA*, *irp2*, and *ybtP* to similar levels as in the wildtype background (Figure 5(a)). We concluded from these results that YbtA contributes moderately as an activator to the yersiniabactin gene expression in *S*. Infantis pESI.

To determine if additional known iron, redox or virulence regulators affect gene expression of the Ybt system in *S*. Infantis, qRT-PCR was conducted, to define the fold change in *fyuA*, *ybtA, irp2*, and *ybtP* expression between the wildtype background and 10 *S*. Infantis null mutants of interest. These gene products were previously reported to control the expression of other virulence factors encoded in pESI (Lrp, ArcA, and ArcB),^23^ regulate gene expression in response to alterations in redox homeostasis (OxyR, SoxR, and FNR),^43^ or orchestrate key virulence phenotypes and stress response in *Salmonella* (RpoS, PhoP, OmpR, and Fur).^44^ Strikingly, these experiments indicated that the expression of *yb*t genes was increased by 100- to 1,000-fold in the absence of Fur (Figure 5 (b-e)). Complementing the activity of Fur from a plasmid (pACYC184::*fur*) reduced the expression of *ybt* genes back to the wildtype levels or below (Figure 5(f)). These results indicated that even though *ybt* genes are encoded on a recently acquired extrachromosomal plasmid, the ancestral regulator, Fur still serves as a key negative regulator that tightly coordinates the expression of this system in *S*. Infantis.

### 3.5. Fur binds directly to the ybtA and irp2 promoters in S. Infantis pESI

**Figure 6.**
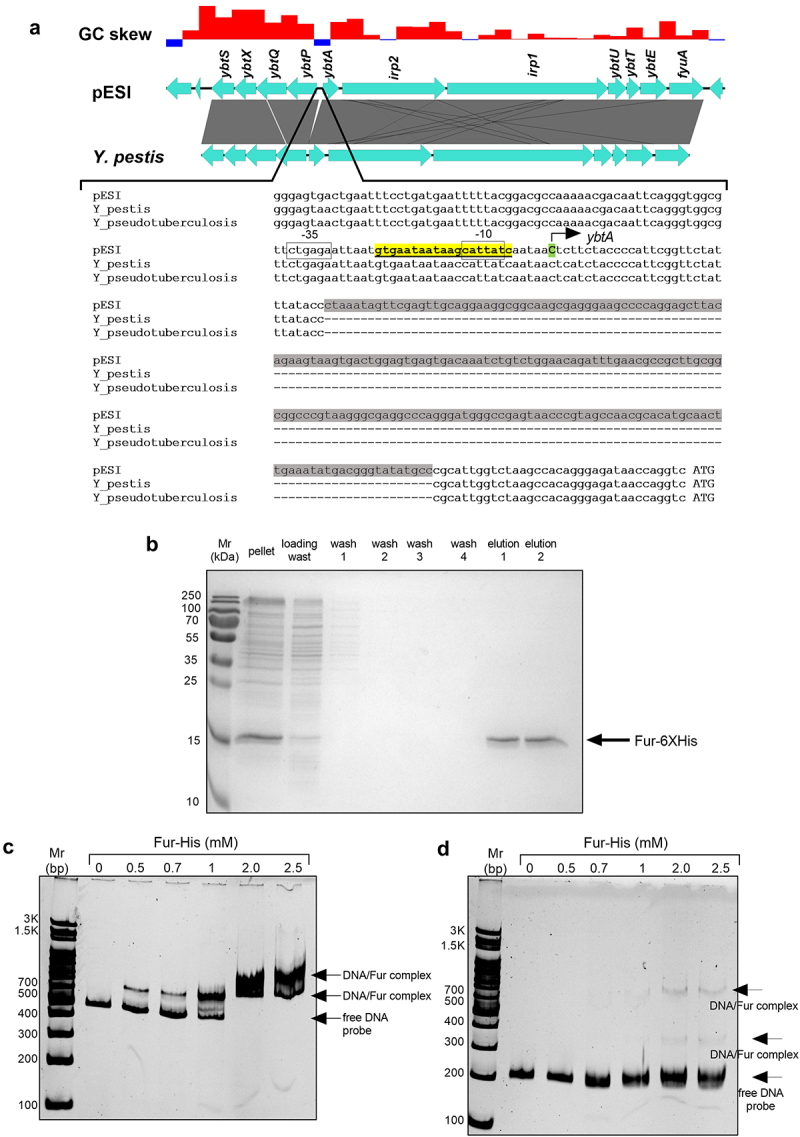
*S*. Infantis Fur binds directly to the *ybtA* promoter in pESI. (a) A pairwise comparison of the yersiniabactin cluster in *S*. Infantis pESI and *Yersinia pestis* was created using the EasyFig tool and is shown at the upper part of the panel. The grey area between the genetic scheme of pESI and *Y.* *pestis* represents the degree of sequence similarity and a 201-bp insertion element in the UTR of *ybtA* in pESI is shown as a white gap in the compared sequences. A histogram showing the GC skew of this region in pESI is shown at the top part of the panel. Multiple sequence alignment of the *ybtA* regulatory region in pESI, *Y.* *pestis* and *Y.* *pseudotuberculosis* is shown in the lower part of the panel. The sequence of the insertion element that is present in pESI, but absent in the *Yersinia spp*. is highlighted in grey. The Fur binding box is highlighted in yellow and the − 10 and − 35 promoter elements are indicated by open boxes. The cytosine, which was determined as the transcriptional start site of *ybtA* in pESI, by RACE is indicated by a capital C and colored in green. (b) The *fur* gene from *S*. Infantis 119944, was cloned into pET28a and introduced into *E.* *coli* BL21 (DE3). Polyhistidine-tagged Fur was induced and purified using a nickel column chromatography. Fractions from each stage of the purification process were separated on 15% polyacrylamide gel electrophoresis that was stained with coomassie blue and imaged. (c) A 378-bp DNA probe corresponding to the regulatory region of *ybtA* in pESI was amplified by PCR and subjected to an electrophoretic mobility shift assay (EMSA). DNA probe (150 ng) was incubated with increasing amounts of purified Fur-His tagged at room temperature and resolved on native 4% polyacrylamide gel. The free DNA probe and gel retardation of DNA-Fur complexes are indicated by arrowheads. (d) A 177-bp DNA probe corresponding to the regulatory region of *ybtA* from *Y.* *Pseudotuberculosis* was amplified by PCR and subjected to an EMSA as in (c). Both gels shown in (b) and (c) were run simultaneously in the same electrophoresis cell.

In *E. coli*, the Fur regulator binds a DNA sequence known as the Fur box. The canonical Fur box sequence is a 9-1-9 inverted repeat of 19-bp composing the sequence 5’-GATAATGATAATCATTATC-3’.^45^ Promoter architecture analysis of the *ybtA-ybtP* intergenic region in pESI using the VirtualFootprint tool^46^ identified a similar 19 bp sequence (5’-**G**TG**AAT**A**ATAA**G**CATTATC-**3’) as a putative Fur binding site (FBS) 272-bp upstream to the start codon of *ybtA*, which also overlaps with the predicted −10 promoter element of *ybtA* (Figure 6(a)). This putative Fur box in pESI is also similar to a previously identified FBSs of *ybtA* in uropathogenic *E. coli* (UPEC),^47^ *Y. pestis* and *Y. enterocolitica*.^48^ Nevertheless, in contrast to the *ybtA* promoter organization in *Yersinia* spp., sequence alignment has identified an insertion element of 201-bp in the *ybtA* intergenic region of pESI (Figure 6(a)). This position was previously identified as one of the three repeat sequences, that serve as YbtA binding sites.^49^ To put these findings in a relevant genetic context, we have applied rapid amplification of cDNA ends (RACE) and determined the transcriptional start site (TSS) of *ybtA*. RACE results indicated that the TSS of *ybA* in *S*. Infantis pESI is a cytosine nucleobase located 5-bp downstream to the FBS and 266-bp upstream to the ATG start codon of *ybtA* (Figure 6(a)).

To further investigate the possibility of a direct Fur binding to the promoter region of *ybtA*, we expressed and purified a recombinant *S*. Infantis His-tagged Fur protein. For this end, the *fur* gene was amplified by PCR from the genome of *S*. Infantis and cloned into pET28a generating an N-terminal hexahistidine tagged Fur. The quality and the purity of the tagged Fur were estimated by sodium dodecyl sulfate polyacrylamide gel electrophoresis (SDS-PAGE) that showed a successful expression and purification of this recombinant Fur (Figure 6(b)). Electrophoretic mobility shift assay (EMSA) analysis was performed following incubation of a 378-bp DNA fragment corresponding to the *ybtA* regulatory region with the His-tagged version of *S*. Infantis Fur in a binding assay. DNA-protein complexes that were resolved on a 4% native polyacrylamide gel showed that in the presence of increasing concentrations of a tagged Fur, the amount of free DNA probe decreased and a prominent retardation in the migration of the DNA-Fur complex appeared in a dose-dependent manner (Figure 6(c)). A control experiment, in which we incubated a 380-bp DNA probe that was amplified from the internal coding sequence of *ybtA* with an increasing amount of Fur-His did not show any retardation in a similar EMSA experiment (Figure S2), indicating that the Fur-His binding to *ybtA* promoter in pESI is specific.

Next, we asked if the presence of the insertion element in the promoter region of *ybtA* in pESI interferes or weakens the binding of Fur to the *ybtA* promoter in pESI. To address that, an EMSA experiment using a DNA probe of a 177-bp, corresponding to the *ybtA* promoter sequence of *Y. pseudotuberculosis* that lacks the pESI insertion element was conducted simultaneously (and in the same gel tank) together with the experiment shown in Figure 6(c). Interestingly, binding of a tagged Fur to a *ybtA* promoter sequence that lacks the insertion was not found to cause more or stronger probe retardation than the binding of Fur to the *ybtA* promoter from pESI (Figure 6(d)), suggesting that the insertion element in the *ybtA* promoter region does not attenuate the ability of Fur to bind to the *ybtA* promoter in pESI, at least under *in vitro* binding conditions.

Similar analyses were also conducted for the promoter region of *irp2*. VirtualFootprint tool identified a putative FBS (**G**T**TAAT**A**AT**T**AT**T**ATT**C**TC**) 44-bp upstream to the *irp2* start codon, which is also overlapped with its predicted −10 promoter element (as predicted by BPROM). EMSA analysis confirmed that His × 6 tagged Fur alone can gel shift the promoter region of *irp2* in a dose-dependent manner (Figure S3). Collectively, these results indicated that the chromosomal encoded *S*. Infantis regulator Fur, can directly and specifically bind to the promoter regions of *ybtA* and *irp2* harbored on the horizontally acquired pESI plasmid in *S*. Infantis.

### 3.6. Ybt contributes to intestinal colonization of S. Infantis in mice, but not to intramacrophage replication

**Figure 7.**
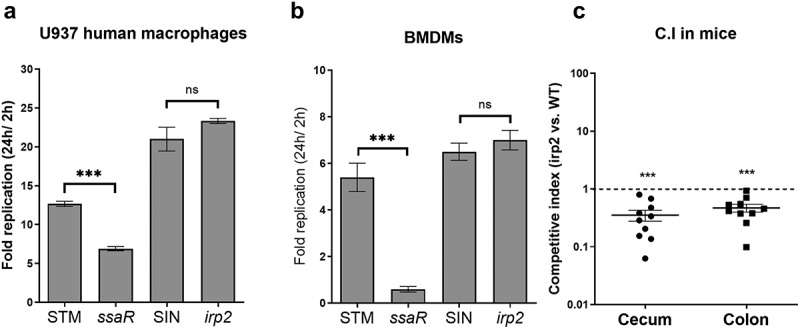
Yersiniabactin is dispensable for intramacrophage growth, but contributes to intestinal colonization of *S*. Infantis in mice. (a-b) Wildtype *S*. Typhimurium (STM), its *ssaR* isogenic mutant (*ssaR*), wildtype *S*. Infantis (SIN), and its isogenic *irp2* mutant (*irp2*) were grown in LB broth to the stationary phase under aerobic conditions at 37°C and used to infect human macrophage U937 (a) and SWISS mice BMDMs (b) at MOI of 10, using the gentamicin protection assay. Intramacrophage replication is shown as the number (CFUs) of intracellular bacteria recovered at 24 h p.i., divided by the number of uptaken intracellular bacteria recovered at 2 h p.i.. Values represent mean and SEM of three independent infections. An unpaired two-tailed student-t test was used to determine statistical differences. (c) Ten female C57BL/6 mice were pretreated with streptomycin by oral gavage 24 h before they were intragastrically infected with 6 × 10^6^ CFU of a mixed (1:1) inoculum containing the wildtype S. Infantis 119944 (harboring pWSK129; Km^R^) and an *irp2* null mutant strain (containing pWSK29; Amp^R^). Three days p.i., mice were euthanized and tissues were harvested aseptically, homogenized and plated onto selective XLD agar plates for bacterial enumeration. Each dot represents a competitive index (CI) value in one mouse in a single organ (cecum or colon). The CI was calculated as [mutant/wildtype] _output_/[mutant/wildtype] _input_. One Sample t-test against a theoretical value of 1 (which indicates equal fitness) was implemented to determine statistical significance. ns, not significant; ***, p < 0.005.

Previously, it was suggested that Ybt limits the availability of iron in activated innate immune cells, which require iron to catalyze the Haber-Weiss reaction to produce hydroxyl radicals needed for generation of an antimicrobial oxidative burst.^50^ To determine whether the Ybt system contributes to *S*. Infantis survival in phagocytic cells, U937 human macrophages as well as mouse bone-marrow-derived macrophages (BMDMs), were infected with *S*. Infantis 119944 wildtype and its *irp2* isogenic strain. In addition, *S*. Typhimurium SL1344 and its *ssaR* derivative mutant strain that is impaired in intracellular replication^51^ were included in this experiment as a positive and negative controls, respectively. As shown in Figure 7(a) and (b), while an *ssaR* mutant was impaired in intracellular replication in comparison to the *S*. Typhimurium wildtype strain, no difference in intramacrophage survival was found between the wildtype *S*. Infantis strain and its isogenic *irp2* mutant, in both tested cell types. We therefore concluded from these experiments that under the above experimental conditions, Ybt does not contribute to *S*. Infantis replication within phagocytic cells, *in vitro*.

To further determine the contribution of the Ybt to *S*. Infantis pathogenicity, we conducted an *in vivo* competition experiment, in which C57BL/6 mice were coinfected with a 1:1 mixed inoculum (6 × 10^6^ CFU) of *S*. Infantis wildtype and its *irp2* isogenic mutant. At 72 h post infection, mice were euthanized and the relative bacterial load of the two strains was determined at different sites. While *S*. Infantis does not cause systemic infection in this mouse model (data not shown), in the cecum and colon of infected mice the *irp2* mutant was out-competed by the wildtype strain by about threefold (Figure 7(c)). These rather modest, but statistically significant differences in colonization suggested that yersiniabactin moderately improves the ability of *S*. Infantis to colonize the intestines of mice, possibly due to better resistance to reactive oxygen species generated as part of the intestinal inflammatory response.

## 4. Discussion

Iron is a critical micronutrient that is required for the function, metabolism and proliferation of all living cells. For pathogenic bacteria, iron acquisition is particularly essential for the survival and persistence during host infection, where soluble ferric iron is absent. To survive under iron-limiting conditions, bacteria have developed miscellaneous strategies to acquire iron, including the production of chelating siderophores with high affinity to oxidized iron (Fe^3+^). Different members of the *Enterobacteriaceae* family including *Yersinia* species synthesize and export the extracellular siderophore yersiniabactin. Genes involved in the production, export and regulation of yersiniabactin are clustered in the high pathogenicity island (HPI), which was proven to confer enhanced virulence of *Yersinia* species,^52^ *Klebsiella pneumoniae*^53^ and several *E. coli* pathotypes.^54^ Moreover, the presence of the yersiniabactin system has been associated with clinical and epidemic strains of Enterobacteriaceae species including uropathogenic *E. coli*,^47^ enteropathogenic *E. coli*,^55^ *Klebsiella pneumoniae*,^56,57^ *Enterobacter hormaechei*^58^ and *S. enterica* serovars.^5,21^

Here, we report that the distribution of yersiniabactin among *Salmonella* genomes is underappreciated and show its presence in at least 10 *S. enterica* serovars (including emerging *S*. Infantis and *S*. Muenchen) from at least three *S. enterica* subspecies, in which the *ybt* cluster could be integrated either as an island in the chromosome or being carried as part of large conjugative plasmids, such as pESI. Previously, we showed that the presence of pESI enhances the fitness of its bacterial host and increases its virulence *in vivo*, however the precise mechanism remained unknown.^5^ In this report, we demonstrate that the *ybt* locus on pESI, is not required for *S*. Infantis growth in iron-limited conditions, but contributes to oxidative stress tolerance and moderately increases *S*. Infantis intestinal colonization in streptomycin pretreated mice. These results indicate that the integration of the yersiniabactin into *S*. Infantis genome, directly contributes to *Salmonella* fitness and its pathogenicity *in vivo*.

Traditionally, functional studies of siderophores have been mainly focused on their role in iron acquisition from the host, but Achard and colleagues have shown that other siderophores (enterobactin and salmochelin) protected *S*. Typhimurium against reactive oxygen species *in vitro*, and allowed better survival after NRAMP^+^ RAW7.5 R macrophage invasion, during the early stage of the infection.^40^ Similarly, other studies have reported that enterobactin protects *E. coli* cells from oxidative stress and that this activity is independent of iron availability.^39^ Here, we show for the first time that yersiniabactin also plays a marked role in hydrogen peroxide tolerance in *Salmonella*. A role for Ybt in oxidative stress tolerance was observed only when bacteria were grown under iron-limiting conditions but not in iron-rich media, when *ybt* genes are suppressed. The fact that a Ybt-dependent protection against hydrogen peroxide was also demonstrated in the absence of iron in a DIP-supplemented medium, implies that the Ybt-mediated protection is most likely independent of its scavenging iron ability, for example by limiting oxidative stress generated through the Fenton reaction that requires ferrous ions. Nevertheless, in contrast to the reported role of enterobactin and salmochelin in *S*. Typhimurium survival in macrophages,^40^ pESI-encoded yersiniabactin was not found to play a detectable role in intramacrophage survival of *S*. Infantis. These results suggest that yersiniabactin may play a different role than enterobactin and salmochelin in macrophage survival of *Salmonella* and that their function is not completely redundant.

In agreement with the demonstrated role of yersiniabactin in oxidative stress response, we were able to show that exposure of *S*. Infantis to sublethal concentrations of hydrogen peroxide led to a significant induction in the transcription of *ybt* genes. Interestingly, the observed upregulation of *ybt* genes was more pronounced in response to oxidative stress than to iron depletion. These results concur with recently reported results showing that the expression of yersiniabactin genes is highly upregulated in response to a hydrogen peroxide treatment in the emerging pathogen *Elizabethkingia anophelis* belonging to the Flavobacteriaceae family.^59^ Taken together, these findings strongly suggest that yersiniabactin plays an overlooked role in response to hydrogen peroxide stress in different pathogens.

As it was shown in other enterobacteriaceae species,^47, 60–62^ the transcriptional regulation of the yersiniabactin genes in pESI-positive *Salmonella* strains is intricate and involved coordinated control by different regulators, including YbtA and the ferric uptake regulator, Fur. Using a reporter gene fusion of the *lux* bioluminescence operon under the promoter of *ybtA* and qRT-PCR approaches we demonstrated induction of *ybtA* under iron-depletion conditions, and in response to oxidative stress during the mid-late logarithmic growth phase of *S*. Infantis. Interestingly, despite the induction of the *ybt* genes in response to hydrogen peroxide, the two signature oxidative stress regulators OxyR and SoxRS^63^ were not found to control the expression of *ybt* genes in *S*. Infantis. However, considering the role of Fur in controlling bacterial defense responses against reactive oxygen species,^64^ it is likely that Fur also orchestrates the induction of *ybt* genes in response to oxidative stress. Indeed, the results reported here about plasmid-borne *ybt* regulation provides an animated example for the regulatory crosstalk between a plasmid-borne regulator (YbtA) and a chromosomally encoded regulator (Fur) and the regulatory assimilation of horizontally acquired genes into the ancestral regulatory setup of *Salmonella*.

In contrast to the *ybtA* 5´ untranslated regions (UTR) architecture in *Y. pestis* and *Y. pseudotuberculosis*, in pESI the *ybtA* UTR was found to include a 201-bp insertion sequence. Interestingly, a different 125-bp Enterobacterial Repetitive Intergenic Consensus (ERIC) element was previously reported to be present in the same position in *Y. enterocolitica* and was suggested to modify the secondary structure of the *ybtA* promoter in this bacterium.^62^ The identification of two distinct insertion elements in the same position in the regulatory region of *ybtA*, in two different species indicates that this locus is a “hot spot” for insertion events that has the potential to affect its regulatory activity due to changes in mRNA stability or binding of corresponding regulators. In this study, we were able to show that the presence of the insertion element in the *ybtA* UTR region, does not prevent and possibly even improves the binding of Fur to this regulatory region. Nonetheless, additional analytical binding experiments and complementary approaches are needed to determine differences in the binding affinity of Fur to this region in the presence and absence of the insertion sequence and to identify potential additional regulators that may bind this DNA element.

Previous studies have shown that salmochelin and enterobactin protect *S*. Typhimurium against reactive oxygen species,^40^ and that catecholate siderophores are important for its full virulence ,^65–67^ and persistent infection,^68^ in the mouse model. A role in virulence was also shown for enterobactin in *S*. Typhi.^69^ The moderately, yet statistically significant, higher colonization of *S*. Infantis wildtype strain compared to the *irp2* mutant in the mice intestines indicates that yersiniabactin may also contribute to *Salmonella* pathogenicity, possibly due to its role in oxidative stress tolerance. It has been shown that during *Salmonella* infection of the gut mucosa, phagocytes recruited during inflammation generate an oxidative burst by releasing reactive oxygen and nitrogen species.^70^ Hence, it is possible that Ybt-positive strains, will be able to better resist the intestinal oxidative stress. Similarly, to these findings with *S*. Infantis, yersiniabactin was also shown to play a role in colonization of uropathogenic *E. coli* (UPEC) in a CBA/J mouse model of ascending urinary tract infection^71^ and in lung colonization by *Klebsiella pneumonia*, using an intranasal infection model in C57BL/6j mice.^53^ Collectively, these studies show that the non-conserved presence of yersiniabactin can enhance the pathogenicity of different Enterobacteriaceae species including *Salmonella* and contribute to the colonization of its bacterial carriers in the context of different infectious diseases.

## 5. Conclusions

The results shown here indicate that the iron-acquisition system, yersiniabactin, which is encoded on the high pathogenicity island of *Yersinia pestis* has an overlooked and variable presence in different *Salmonella* genomes. The *ybt* cluster could be integrated either in the chromosome or being carried by different conjugative plasmids, including pESI. Interestingly, despite the presence of an insertion sequence in the promoter of *ybtA* in pESI, *ybt* genes are tightly regulated by YbtA and Fur, which binds directly to the *ybtA* and *irp2* promoters. Furthermore, we found that pESI-encoded *ybt* genes are specifically induced during the logarithmic growth phase of *S*. Infantis cultures and under iron-depriving conditions or in response to hydrogen peroxide. Interestingly, the yersiniabactin system was found to be dispensable for *S*. Infantis growth under iron-depleted conditions, but to play a profound role in oxidative stress tolerance and to contribute moderately to intestinal colonization of *S*. Infantis in the acute salmonellosis mouse model. These results indicate that the integration of the yersiniabactin contribute to *Salmonella* fitness and its pathogenicity *in vivo* and may help to explain the rapid emergence of pESI in different epidemic *Salmonella* strains.

## Supplementary Material

Supplemental Material

## Data Availability

The authors confirm that the data supporting the findings of this study are available within the article and its supplementary materials.
