# Supplementary material for: The transcriptional regulation of the horizontally acquired iron uptake system, yersiniabactin and its contribution to oxidative stress tolerance and pathogenicity of globally emerging salmonella strains
Source: Gut Microbes. 2024 Jul 4;16(1):2369339. doi: 10.1080/19490976.2024.2369339 (PMC11225919; doi:10.1080/19490976.2024.2369339)
Supplement: Supplemental Material [file KGMI_A_2369339_SM4476.zip › Supplementary_materials_R1.docx]

**Supplementary materials**

**Supplementary Figures**

**Supplementary Figure S1. Yersiniabactin contributes to oxidative stress tolerance under iron-depleted conditions.** *S*. Infantis 119944, its isogenic *irp2* null mutant, and a complemented *irp2* mutant strain harboring pWSK29::*irp2* or the empty vector (pWSK29) were grown in LB medium and subcultured 1:30 into M9 medium supplemented with 50 µM DIP and grown at 37°C with shaking until reaching OD_600_ of ~1. Hydrogen peroxide at a final concentration of 40 mM was added to the cultures, which were further incubated at 37°C. At 20 and 30 min post H_2_O_2_ challenging, serial dilutions were plated on LB agar plates to determine the number of viable CFUs relative to time 0. The graph shows the mean of three biological repeats with SEM indicated by the error bars. One-way ANOVA was used to test statistical significance. ns, not significant; ***, P<0.005.

**Supplementary Figure S2. The binding of 6×His-Fur to *ybtA* promoter is specific**. Two DNA probes of 378 and 380-bp corresponding to the promoter region of *ybtA* in pESI and part of its coding sequence (CDS), respectively were amplified by PCR and subjected to an electrophoretic mobility shift assay (EMSA). Following incubation of the DNA probes (150 ng each) in a binding buffer in the presence of increasing amount of purified Fur-His tagged (0, 0.7, 1, and 2 mM). The DNA-protein complexes were resolved on native 4% polyacrylamide gel, stained with GelRed and imaged. The free DNA probes and gel retardation of DNA-Fur complexes are indicated by arrowheads.

**Supplementary Figure S3. Fur binds directly to the *irp2* promoter in pESI**. (**A**) A genetic map of the *ybt* locus in pESI is shown at the top section of the panel. The sequence of the *irp2* regulatory region is shown below, highlighting the Fur binding box in yellow. The putative -10 and -35 promoter elements as predicted by the BPROM tool are framed by open boxes. (**B**) A 190-bp DNA probe corresponding to the regulatory region of *irp2* in pESI was amplified by PCR and subjected to an electrophoretic mobility shift assay (EMSA). DNA probe (150 ng) was incubated with increasing amounts of purified His-tagged Fur at room temperature in a binding buffer. The DNA-protein complexes were resolved on native 4% polyacrylamide gel, stained with GelRed and imaged. The free DNA probe and gel retardation of a DNA-Fur complexes are indicated by arrowheads

**Supplamentary tables**

**Table S1. Bacterial strains used in this study**

| **Strains** | **Description** | **Source or reference** |
| --- | --- | --- |
| *S*. Infantis 119944 | 2008 clinical isolate of emerging *S*. Infantis harboring pESI | ^1^ |
| *S*. Infantis 119944 *irp2* | In-frame *irp2* deletion mutant in *S*. Infantis 119944 | This study |
| *S*. Infantis 119944 *ybtA* | In-frame *ybtA* deletion mutant in *S*. Infantis 119944 | This study |
| *S*. Infantis 119944 *entC* | In-frame *entC* deletion mutant in *S*. Infantis 119944 | This study |
| *S*. Infantis 119944 *iroB* | In-frame *iroB* deletion mutant in *S*. Infantis 119944 | This study |
| *S*. Infantis 119944 *iroBirp2* | In-frame *iroB* and *irp2* deletion mutant in *S*. Infantis 119944 | This study |
| *S*. Infantis 119944 *iroBentC* | In-frame *iroB* and *entC* double deletion mutant in *S*. Infantis 119944 | This study |
| *S*. Infantis 119944 *irp2iroBentC* | In-frame *iroB*, *entC* and *irp2* triple deletion mutant in *S*. Infantis 119944 | This study |
| *S*. Infantis 119944 *fnr* | In-frame *fnr* null deletion in *S*. Infantis 119944 | ^2^ |
| *S*. Infantis 119944 *fur* | In-frame *fur* null deletion in *S*. Infantis 119944 | ^2^ |
| *S*. Infantis 119944 *arcA* | In-frame *arcA* null deletion in *S*. Infantis 119944 | ^2^ |
| *S*. Infantis 119944 *arcB* | In-frame *arcB* null deletion in *S*. Infantis 119944 | ^2^ |
| *S*. Infantis 119944 *phoP* | In-frame *phoP* null deletion in *S*. Infantis 119944 | ^2^ |
| *S*. Infantis 119944 *ompR* | In-frame null deletion in *S*. Infantis 119944 | ^2^ |
| *S*. Infantis 119944  *oxyR* | In-frame *oxyR* null deletion in *S*. Infantis 119944 | ^2^ |
| *S*. Infantis 119944 *soxRS* | In-frame *soxRS* null deletion in *S*. Infantis 119944 | ^2^ |
| *S*. Infantis 119944 *lrp* | In-frame *lrp* null deletion in *S*. Infantis 119944 | ^2^ |
| *S*. Infantis 119944 *phoP* | In-frame *phoP* null deletion in *S*. Infantis 119944 | ^2^ |
| *S*. Infantis 119944 *rpoS* | In-frame *rpoS* null deletion in *S*. Infantis 119944 | ^2^ |
| *S*. Typhimurium SL1344 | wild type Sm^r^ *xyl hisG rpsL* | SGSS |
| S. Typhimurium SL1344 *ssaR* | *ssaR* deletion mutant in *S*. Typhimurium SL1344 | ^3^ |
| *E. coli* DH5α |  | Lab collection |
| *E. coli* BL21 (DE3) |  | Lab collection |
| **plasmids** |  |  |
| pKD46 | Temperature-sensitive origin of replication and encodes inducible Red recombinase | ^4^ |
| pKD3 | template plasmid for frt-flanked cat (Cm^r^) cassette | ^4^ |
| pCP20 | Temperature-sensitive origin of replication, confers ampicillin resistance, and encodes the FLP recombinase | ^4^ |
| pWSK29 | Amp^r^ low copy number cloning vector | ^5^ |
| pWSK129 | Km^r^ low copy number cloning vector | ^5^ |
| pCS26 | Kan^r^ cloning vector carrying the *luxCDABE* operon of the bacterium *Photorhabdus luminescens* | ^6^ |
| pET28a | Bacterial vector for expression of N-terminally 6xHis-tagged protein | - Lab collection |
| pET28a::fur | *S*. Infantis 119944 *fur* cloned into pET28a | This study |
| pWSK29::*irp2* | *S*. Infantis 119944 (pESI) *irp2* cloned into pWSK29 | This study |
| pCS26::P*ybtA* | *S*. Infantis 119944 (pESI) *ybtA* promoter cloned into pWSK29 | This study |
| pWSK29::*ybtA* | *S.* Infantis 119944 (pESI)  *ybtA* cloned into pWSK29 under its native promoter | This study |
| pWSK29::*entC* | *S.* Infantis 119944 *entC* cloned into pWSK29 under its native promoter | This study |
| pACYC184::*fur* | *S*. Infantis 119944 *fur* gene cloned into pWSK29 under its native promoter | ^7^ |

**Table S2. Primers used in this study**

| **Primer name** | **Sequence (5' to 3')** | **Purpose** |
| --- | --- | --- |
| rpoD_Fw | CGCCAGATGCTGCAAGAGAT | RT-PCR of *rpoD* |
| rpoD_Rev | CCGATCGGCGTTTCCATGG |  |
| irp2- FW- RT | GCAGGCACAGCTTGATGATA | RT-PCR of *irp2* |
| irp2- rev- RT | TTGACCACACCGACAACAGT |  |
| ybtA- FW-RT | CAAGATCGACTCCCAATGCT | RT-PCR of *ybtA* |
| ybtA- Rv-RT | TCATCCAGCACCAGACTGAG |  |
| ybtP- RT_ FW | TGTAACCTTCTGGCAACACG | RT-PCR of *ybtP* |
| ybtP- RT_ Rv | AGACGGACACCCTGATGAAG |  |
| psn- RT- Fw | TCACAGCAAGACGAAAGCAC | RT-PCR of *psn* (*fyuA*) |
| psn- RT- Rv | TATTGAGCCCAGGCAAGACT |  |
| oxyR-RT-Fw | GGTATCTTCATCCGCTCCCG | RT-PCR of *oxyR* |
| oxyR-RT- Rv | CGTTTATCGAAGTGCCGCTG |  |
| Fur for pet28a Nde1 Fw | AAACATATGACTGACAACAATACCGCA | Fur cloning in pET28a |
| Fur BamH1 Rv- new | AAAAGGATCCTTATTTAGTCGCGTCATCGT |  |
| ybtA pro_ IP 32953_Fw | GGAGTAACTGAATTTCCTGATG | DNA probe of *ybtA* |
| ybtA pro_119944_Fw | GGAGTGACTGAATTTCCTGATG |  |
| ybtA pro_SIN+yersi_Rv | CGGTGACTCCGTCATGACCTGG | PCR of *ybtA* regulatory region |
| irp2 middle Fw | GTGGGTTGCCACCTGTATCA | PCR probe from *irp2* CDS |
| irp2 middle Rv | CTTTCAGCGTCGTTATGGCG |  |
| ybtA middle Fw | ATCCTGGAAGGCACATCACG | PCR probe from *ybtA* coding region |
| ybtA middle Rv | AGCACCAGACTGAGCATGAC |  |
| irp2 promoter_Fw | CCGGGGTCGCGCCCCCATAA | PCR probe of *irp2* promoter |
| irp2 promoter_Rv | TCTTCCTCCTGATGGCACGTC |  |
| ybtA-KO_Fw | TCTAAGCCACAGGGAGATAACCAGGTCATGACGGAGTCACCGCAAGTGTAGGCTGCTTCG | *ΔybtA* mutant |
| ybtA-KO_Rv- | AGGCGTTTATGGGGGCGCGACCCCGGTTACATCACGCGTTTAAAGGTATACTTATAGGAGGAAT |  |
| iroB KO Fw | TTATGACGTGGAGAGAGAGGATTTCTCATGCGTATTCTGTTTGTCGTGTAGGCTGGAGCTGCTTCG | *ΔiroB* mutant |
| iroB KO Rv | CGCCATATTGTCATTGCGCTGCCGCGGTTAGCCGTGTTGCAGCATCATATGAATATCCTCCTTA |  |
| iroB seq Fw | GACCATACGCGCGTAAATAA | *ΔiroB* sequence verification |
| iroB seq Rv | GGTCGATACTGATGCTACGA |  |
| P1- entC KO | TTCATGTCAGCGGCAGCGAG | *ΔentC* mutant |
| P2- entC KO | GAAGCAGCTCCAGCCTACACACATATCCATATCATCCTCCA | *ΔentC* mutant |
| P3- entC KO | GATATACAGTTCCGGTACAT | *ΔentC* mutant |
| P4- entC KO | CTAAGGAGGATATTCATATGGGACTGCATTAAGGAACGAT | *ΔentC* mutant |
| entC seq Fw | TCATTCAAGAAGCATCGCGA | *ΔentC* sequence verification |
| entC seq Rv | CGGTTTCGCCAGGTTTGATG | *ΔentC* sequence verification |
| ybtA gene-specific primer | GATTACGCCAAGCTTGCTTCCTGCATTCGTTCAGCCTGAA | *ybtA* RACE |
| ybtA middle Rv | AGCACCAGACTGAGCATGAC | *ybtA* RACE sequencing |
| Fw ybtA prom pCS26 | aaaa**CTCGAG**GGGAGTGACTGAATTTCCTG | Cloning *ybtA* promoter in pCS26 |
| Rev ybtA prom pCS26' | aaaa**GGATCC**GACCTGGTTATCTCCCTGTG |  |
| irp2 fw Hind3 | aaaaaA**AGCTT**TCATGACGGTGGCAGCCGA | *irp2* cloning in pWSK29 |
| irp2 rev BamH1 | aaaaa**GGATCC**CTATATCCGCCGCTGACGAC |  |
| ybtA_fwd_Comp | gaacaaaagctggagctccaccgcggtggcggccgcTTACATCACGCGTTTAAAGG | *ybtA* cloning in pWSK29 |
| ybtA_rev_Comp | agcttgatatcgaattcctgcagcccgggggatccGGGAGTGACTGAATTTCCTG |  |
| entC_COMP_FW_bamHI | AAAAAAGGATCCAATAGCGTCCTGTTATTAAT | entC cloning in pWSK29 |
| entC_COMP_REV_sac1 | AAAAAAGAGCTCCTTAATGCAGTCCAAAAACG |  |

**Table S3**. The degree of identity and similarity between Ybt proteins in *Y. pestis* and their homologs in *Salmonella*.

| *Y. pestis* | **YbtA** | **Irp2** | **Irp1** | **YbtU** | **YbtT** | **YbtE** | **FyuA** |
| --- | --- | --- | --- | --- | --- | --- | --- |
| S. Infantis | Identity:  315/320 (98%)  Similarity:  317/320 (99%) | Identity: 1982/2029 (98%)  Similarity: 2000/2029 (98%) | Identity:  3079/3166 (97%)  Similarity: 3111/3166 (98%) | Identity: 359/366 (98%)  Similarity: 361/366 (98%) | Identity:  257/263 (98%)  Similarity: 258/263 (98%) | Identity: 511/526 (97%)  Similarity: 519/526 (98%) | Identity: 657/674 (97%)  Similarity: 665/674 (98%) |
| S. Typhimurium | Identity: 315/320 (98%)  Similarity: 317/320 (99%) | Identity: 1982/2029 (98%)  Similarity: 2000/2029 (98%) | Identity: 3079/3166 (97%)  Similarity: 3111/3166 (98%) | Identity: 360/366 (98%)  Similarity: 362/366 (98%) | Identity: 257/263 (98%)  Similarity: 258/263 (98%) | Identity: 511/526 (97%)  Similarity: 519/526 (98%) | Identity: 657/674 (97%)  Similarity: 665/674 (98%) |
| S. Newport | Identity: 315/320 (98%)  Similarity: 317/320 (99%) | Identity: 1982/2029 (98%)  Similarity: 2000/2029 (98%) | Identity: 3079/3166 (97%)  Similarity: 3111/3166 (98%) | Identity: 360/366 (98%)  Similarity: 362/366 (98%) | Identity: 257/263 (98%)  Similarity: 258/263 (98%) | Identity: 511/526 (97%)  Similarity: 519/526 (98%) | Identity: 657/674 (97%)  Similarity: 665/674 (98%) |
| S. Minnesota | Identity: 315/320 (98%)    Similarity: 317/320 (99%) | Identity: 1982/2029 (98%)  Similarity: 2000/2029 (98%) | Identity: 3078/3166 (97%)  Similarity: 3110/3166 (98%) | Identity: 360/366 (98%)  Similarity: 362/366 (98%) | Identity: 257/263 (98%)  Similarity: 258/263 (98%) | Identity: 511/526 (97%)  Similarity: 519/526 (98%) | Identity: 657/674 (97%)  Similarity: 665/674 (98%) |
| S. Macclesfield | Identity: 315/320 (98%)  Similarity: 317/320 (99%) | Identity: 1982/2029 (98%)  Similarity: 2000/2029 (98%) | Identity: 3080/3166 (97%)  Similarity: 3113/3166 (98%) | Identity: 359/366 (98%)  Similarity: 361/366 (98%) | Identity: 258/263 (98%)  Similarity: 259/263 (98%) | Identity: 511/526 (97%)  Similarity: 519/526 (98%) | Identity: 658/674 (98%)  Similarity: 666/674 (98%) |
| S. Heidelberg | Identity: 315/320 (98%)  Similarity: 317/320 (99%) | Identity: 1982/2029 (98%)  Similarity: 2000/2029 (98%) | Identity: 3079/3166 (97%)  Similarity: 3111/3166 (98%) | Identity: 360/366 (98%)  Similarity: 362/366 (98%) | Identity: 257/263 (98%)  Similarity: 258/263 (98%) | Identity: 511/526 (97%)  Similarity: 519/526 (98%) | Identity: 657/674 (97%)  Similarity: 665/674 (98%) |
| *S. enterica* subsp. diarizonae | Identity: 315/320 (98%)  Similarity: 316/320 (98%) | Identity: 1987/2029 (98%)  Similarity: 2003/2029 (98%) | Identity: 3089/3164 (98%)  Similarity: 3116/3164 (98%) | Identity: 360/366 (98%)  Similarity: 362/366 (98%) | Identity: 258/263 (98%)  Similarity: 259/263 (98%) | Identity: 514/526 (98%)  Similarity: 520/526 (98%) | Identity: 657/674 (97%)  Similarity: 667/674 (98%) |
| *S. enterica* subsp. salamae | Identity: 315/320 (98%)  Similarity: 317/320 (99%) | Identity: 1982/2029 (98%)  Similarity: 2000/2029 (98%) | Identity: 3079/3166 (97%)  Similarity: 3111/3166 (98%) | Identity: 360/366 (98%)  Similarity: 362/366 (98%) | Identity: 257/263 (98%)  Similarity: 258/263 (98%) | Identity: 511/526 (97%)  Similarity: 519/526 (98%) | Identity: 657/674 (97%)  Similarity: 665/674 (98%) |

**References**

1. Cohen E, Rahav G, Gal-Mor O. Genome Sequence of an Emerging Salmonella enterica Serovar Infantis and Genomic Comparison with Other S. Infantis Strains. Genome Biol Evol 2020; 12:151-9.

2. Aviv G, Rahav G, Gal-Mor O. Horizontal Transfer of the Salmonella enterica Serovar Infantis Resistance and Virulence Plasmid pESI to the Gut Microbiota of Warm-Blooded Hosts. mBio 2016; 7.

3. Brumell JH, Rosenberger CM, Gotto GT, Marcus SL, Finlay BB. SifA permits survival and replication of Salmonella typhimurium in murine macrophages. Cell Microbiol 2001; 3:75-84.

4. Datsenko KA, Wanner BL. One-step inactivation of chromosomal genes in Escherichia coli K-12 using PCR products. Proc Natl Acad Sci U S A 2000; 97:6640-5.

5. Wang RF, Kushner SR. Construction of versatile low-copy-number vectors for cloning, sequencing and gene expression in *Escherichia coli*. Gene 1991; 100:195-9.

6. Bjarnason J, Southward CM, Surette MG. Genomic profiling of iron-responsive genes in Salmonella enterica serovar typhimurium by high-throughput screening of a random promoter library. J Bacteriol 2003; 185:4973-82.

7. Aviv G, Elpers L, Mikhlin S, Cohen H, Vitman Zilber S, Grassl GA, et al. The plasmid-encoded Ipf and Klf fimbriae display different expression and varying roles in the virulence of Salmonella enterica serovar Infantis in mouse vs. avian hosts. PLoS Pathog 2017; 13:e1006559.
