# Supplementary figures and images for: The transcriptional regulation of the horizontally acquired iron uptake system, yersiniabactin and its contribution to oxidative stress tolerance and pathogenicity of globally emerging salmonella strains
Source: Gut Microbes. 2024 Jul 4;16(1):2369339. doi: 10.1080/19490976.2024.2369339 (PMC11225919; doi:10.1080/19490976.2024.2369339)

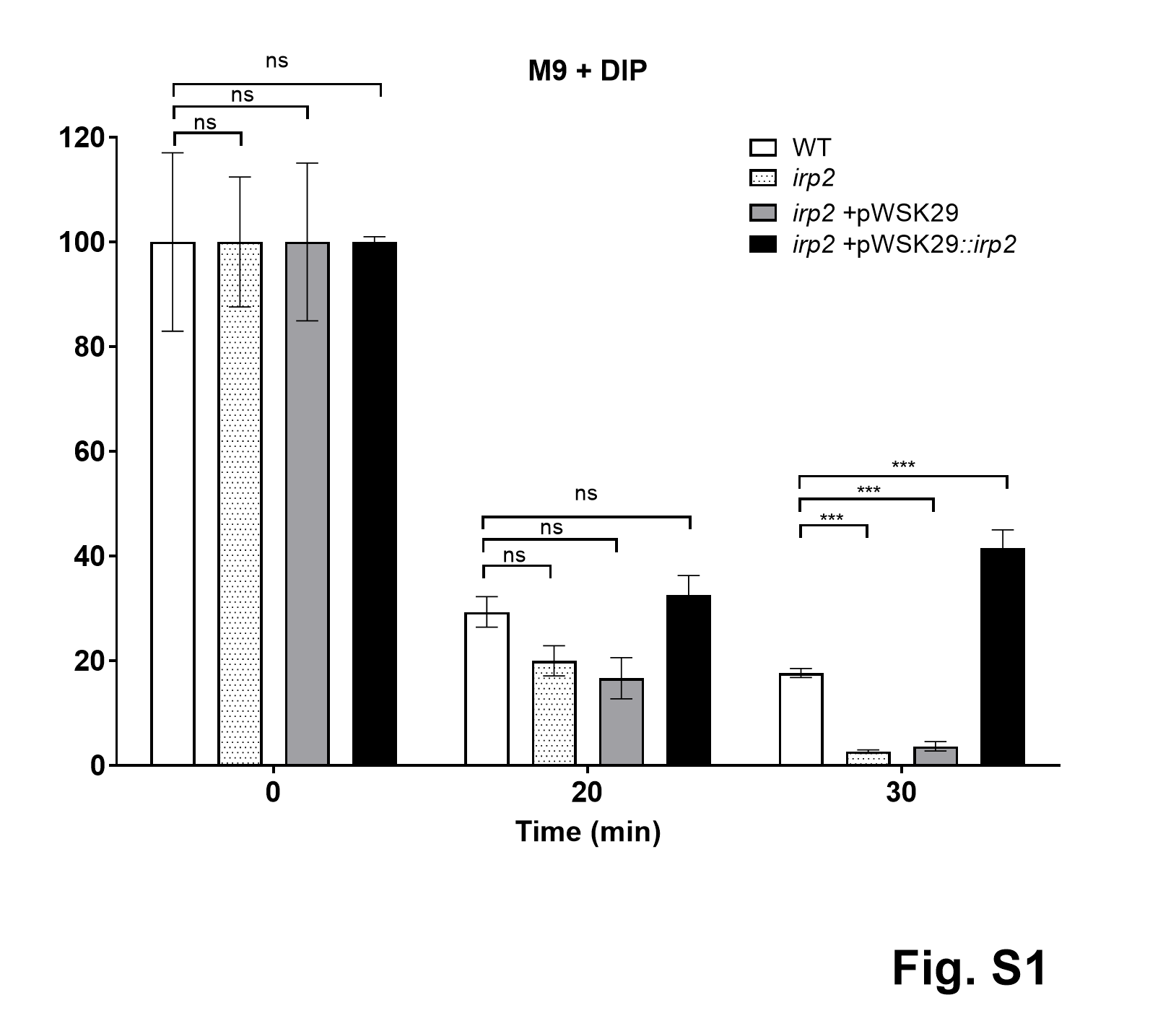

Supplement: Supplemental Material [file KGMI_A_2369339_SM4476.zip › Fig_S1.tif]

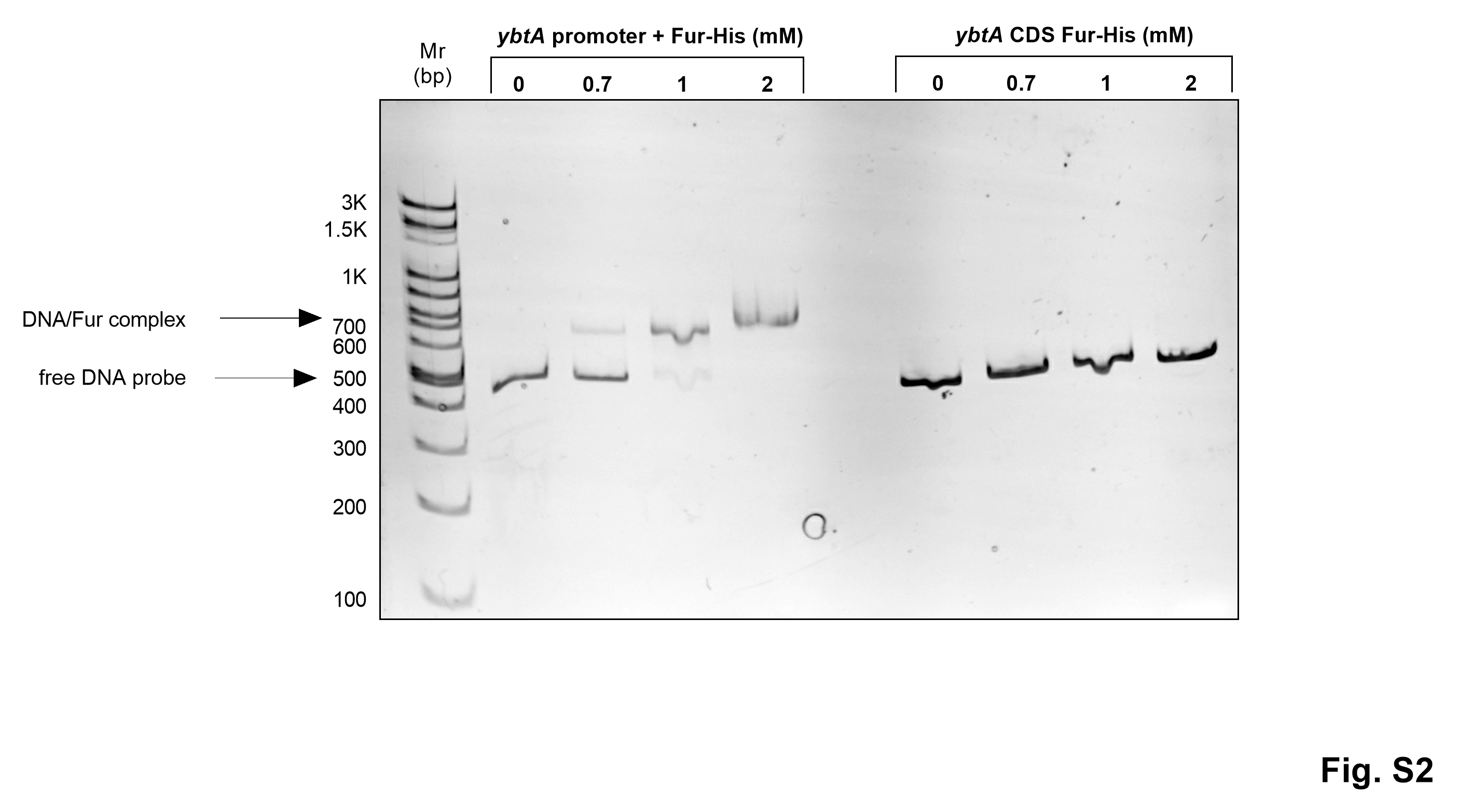

Supplement: Supplemental Material [file KGMI_A_2369339_SM4476.zip › Fig_S2.tif]

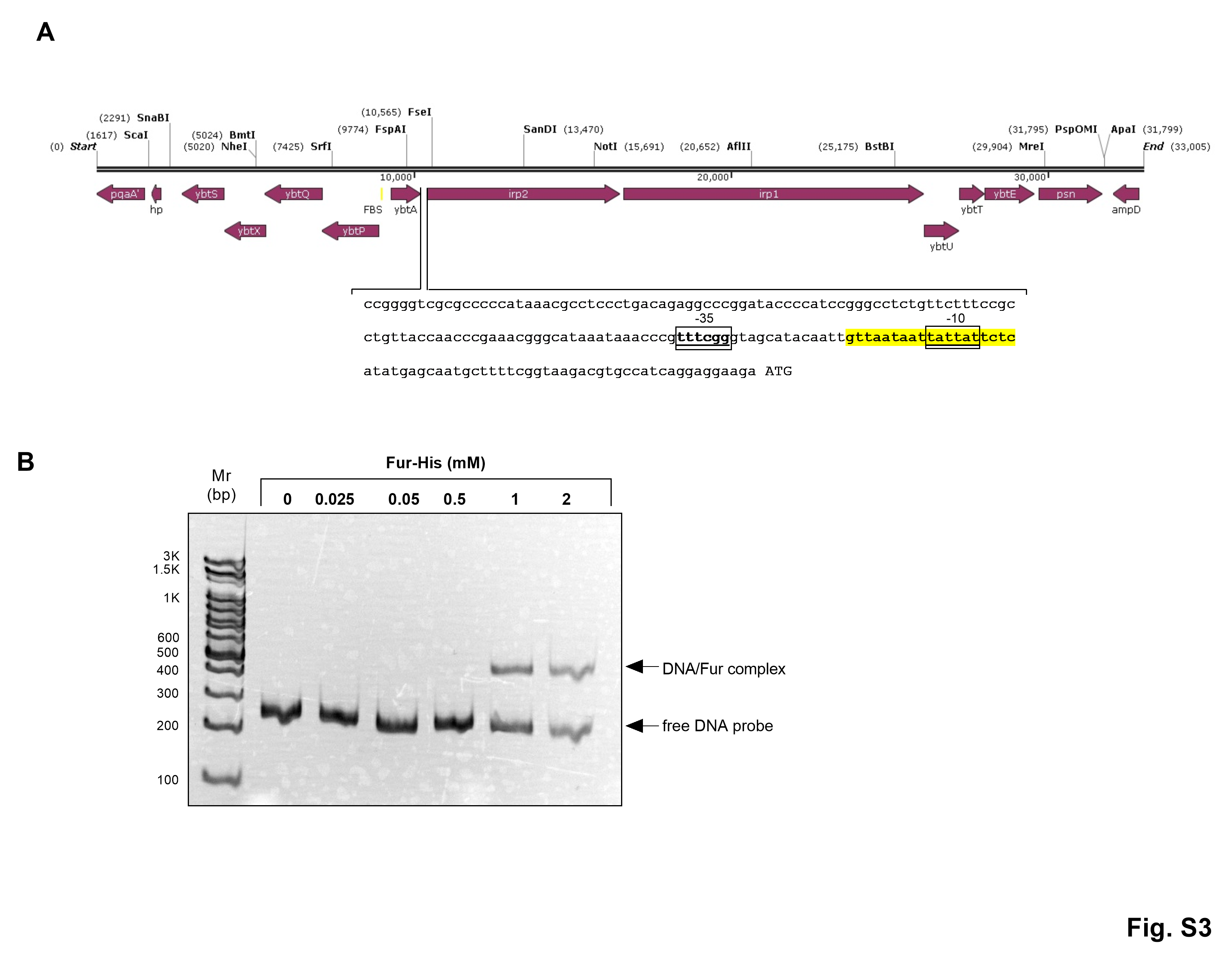

Supplement: Supplemental Material [file KGMI_A_2369339_SM4476.zip › Fig_S3 (1).tif]
